# Prion Uptake in the Gut: Identification of the First Uptake and Replication Sites

**DOI:** 10.1371/journal.ppat.1002449

**Published:** 2011-12-22

**Authors:** Pekka Kujala, Claudine R. Raymond, Martijn Romeijn, Susan F. Godsave, Sander I. van Kasteren, Holger Wille, Stanley B. Prusiner, Neil A. Mabbott, Peter J. Peters

**Affiliations:** 1 Section of Cell Biology II, Netherlands Cancer Institute, Amsterdam, The Netherlands; 2 The Roslin Institute and Royal (Dick) School of Veterinary Sciences, University of Edinburgh, Easter Bush, Midlothian, United Kingdom; 3 Institute for Neurodegenerative Diseases and Department of Neurology, University of California, San Francisco, California, United States of America; 4 Kavli Institute of Nanoscience, Delft University of Technology, Delft, The Netherlands; Istituto Superiore di Sanità, Italy

## Abstract

After oral exposure, prions are thought to enter Peyer's patches via M cells and accumulate first upon follicular dendritic cells (FDCs) before spreading to the nervous system. How prions are actually initially acquired from the gut lumen is not known. Using high-resolution immunofluorescence and cryo-immunogold electron microscopy, we report the trafficking of the prion protein (PrP) toward Peyer's patches of wild-type and PrP-deficient mice. PrP was transiently detectable at 1 day post feeding (dpf) within large multivesicular LAMP1-positive endosomes of enterocytes in the follicle-associated epithelium (FAE) and at much lower levels within M cells. Subsequently, PrP was detected on vesicles in the late endosomal compartments of macrophages in the subepithelial dome. At 7–21 dpf, increased PrP labelling was observed on the plasma membranes of FDCs in germinal centres of Peyer's patches from wild-type mice only, identifying FDCs as the first sites of PrP conversion and replication. Detection of PrP on extracellular vesicles displaying FAE enterocyte-derived A33 protein implied transport towards FDCs in association with FAE-derived vesicles. By 21 dpf, PrP was observed on the plasma membranes of neurons within neighbouring myenteric plexi. Together, these data identify a novel potential M cell-independent mechanism for prion transport, mediated by FAE enterocytes, which acts to initiate conversion and replication upon FDCs and subsequent infection of enteric nerves.

## Author Summary

Prion diseases are orally transmissible, but how the abnormally folded isoform of the prion protein (PrP^Sc^) transits from the gastrointestinal tract to infect neural tissues is not known. Here we demonstrate that in contrast to the current literature, PrP^Sc^ enters Peyer's patches primarily through specialised enterocytes with much lower levels trafficking through M cells. Proteins from homogenized PrP^Sc^ infected brain tissue are transcytosed across the follicle-associated epithelium and delivered to macrophages and follicular dendritic cells, which appear to serve as the primary site of PrP conversion and replication following oral exposure to PrP^Sc^ before infecting the enteric nerves.

## Introduction

Prions are infectious proteins composed of an abnormally folded isoform of the prion protein (PrP^Sc^), the accumulation of which causes variant Creutzfeldt–Jakob disease, scrapie, and bovine spongiform encephalopathy, among other diseases. Prions propagate by converting endogenous, cellular prion protein (PrP^C^) into PrP^Sc^ containing a β-sheet core. Isolated PrP^Sc^ can be found in a wide range of aggregation states, from small oligomers to amyloid, and at least in larger aggregates the C-terminal portion of PrP^Sc^ acquires resistance to protease treatment [1]. PrP^C^ is a ubiquitously expressed protein that is most abundant in the nervous system. The accumulation of PrP^Sc^ causes morphological changes in the central nervous system including astrocytosis, neuronal cell loss and spongiform pathology and, in some types of prion disease, amyloid plaque formation. Pathology builds up during a long incubation period that ends in a short clinical phase and death. Expression of PrP^C^ in the host is required for successful infection, since it provides the substrate for the conversion to PrP^Sc^ [2]–[5].

Prions are highly resistant to denaturation by chemical and physical means, making disposal and disinfection difficult. This resistance may also contribute to their ability to survive passage through the digestive tract [6], allowing transmission of prion disease via prion-contaminated food. Many naturally occurring prion diseases are considered to be acquired orally, and are accompanied by accumulation of PrP^Sc^ in the lymphoreticular system long before invasion of the nervous system takes place [7]–[13]. Indeed, when specific components of the gut-associated lymphoid tissues (GALT) are absent, the transport of prions from the gut lumen to the nervous system is dramatically impaired [9], [12], [14]. The exact mechanisms by which infectious prions are transmitted from the gut lumen to the central nervous system remain elusive (for reviews see [15]–[17]).

The luminal surface of the intestine limits the access of pathogenic microorganisms to the underlying host tissues, and is protected by a single layer of epithelial cells bound by tight-junctions. Located within the villus epithelium and follicle-associated epithelium (FAE) of the Peyer's patch are microfold cells (M cells), a unique epithelial cell type specialized for the transepithelial transport of macromolecules and particles (for a review of M cells see [18]). M cells enable the host's immune system to sample the intestinal lumen and mount an appropriate immune response. However, some pathogenic microorganisms exploit M cells and use them to gain entry into mucosal tissues [18]. Using an *in vitro* system, M cell-like cells have been shown to actively transcytose the scrapie agent through to the basolateral side of the epithelium [19], [20], and studies in mice suggest prions might likewise be translocated across the FAE by M cells *in vivo* [21].

Together these data imply that M cells are plausible sites for the transepithelial transport of TSE agents across the intestinal epithelium. However, other data suggest such transport might also occur independently of M cell-mediated transcytosis via enterocytes [22], [23]. Studies in which isolated sheep gut loops were injected with scrapie brain homogenate [22] suggested that disease-specific PrP was transported across the absorptive epithelium of villi into lacteals. Other studies have shown that in response to inflammatory stimuli, mononuclear phagocytes within the lamina propria, including macrophages and classical dendritic cells (DC), can insert dendrites through the tight junctions between intestinal epithelial cells. These projections enable the cells to sample the luminal contents directly [24] implying another potential route of transepithelial TSE agent transport. Clearly, the way that orally introduced, partially proteinase-resistant prions survive the proteolytic conditions in the alimentary tract without losing all their infectivity and then cross the gut epithelium is still a matter of debate.

After passing the epithelial barrier, prions are thought to be captured by underlying migratory classical dendritic cells [25] that transmit the infectious agents by an as-of-yet unknown mechanism to the germinal centres. Within the germinal centres, prions accumulate upon follicular dendritic cells (FDCs), which are specialized stromal, mesenchymal cells of the immune system. FDCs are nonphagocytic, nonmigratory cells that trap native immune complexes on their surface through the expression of cellular complement receptors. The expression of high levels of PrP^C^ in FDCs is regarded to be important for the accumulation and replication of prions upon their surfaces [26]–[30].

FDCs are considered to amplify the prions above the threshold level necessary to infect peripheral nerves [9], [12], [14], [31]. Prions are then thought to gain access to the central nervous system via physical interaction with peripheral nerve fibers of the enteric nervous system [32]. Retrograde axonal transport would deliver prions to their main pathological target, the brain. Due to the lack of satisfactory experimental models, some of the observations in prion transmission are based solely on *in vitro* studies.

Here, we examined the *in vivo* time-course of oral prion infection in the GALT of wild-type (wt) mice that are susceptible to prion infection and of PrP-deficient (*Prnp* ^–/–^) mice that do not succumb to prion disease [33]. Both wt and *Prnp* ^–/–^ mice were orally infected with 3 different rodent-adapted scrapie strains (ME7, RML and Sc327), and intestinal and lymphoid tissues collected at specific days post-feeding (dpf). Samples were then analysed by immunofluorescence (IF) and cryo-immunogold electron microscopy (cryo-immuno EM) in order to identify, at the ultrastructural level, the cell types and subcellular organelles that are involved in prion trafficking and early pathogenesis. Our data show that prion uptake and transfer across the follicle-associated epithelium (FAE) of the gut occurred of the follicle-associated epithelium (FAE) independently of cellular PrP^C^ expression. We show that PrP was first transiently detectable mainly within large LAMP1-positive endosomes of FAE enterocytes and at much lower levels within M cells. Proteins of FAE enterocytes were found on vesicles in the extracellular material adjacent to FAE enterocytes, and on the surface of FDCs, and these vesicles could act to transport prions towards FDCs. Furthermore, between 7–21 dpf, increased PrP labelling was only observed on the plasma membranes of FDCs from wild-type mice, identifying these membranes as the first site of PrP conversion and replication within Peyer's patches. Together, these data identify a novel potential M cell-independent route of prion uptake and transfer from the gut lumen mediated by FAE enterocytes that may have an important influence on susceptibility to oral prion infection.

## Results

### Enterocytes within the FAE contain unique large apical LAMP1-positive endosomes

**Figure 1:**
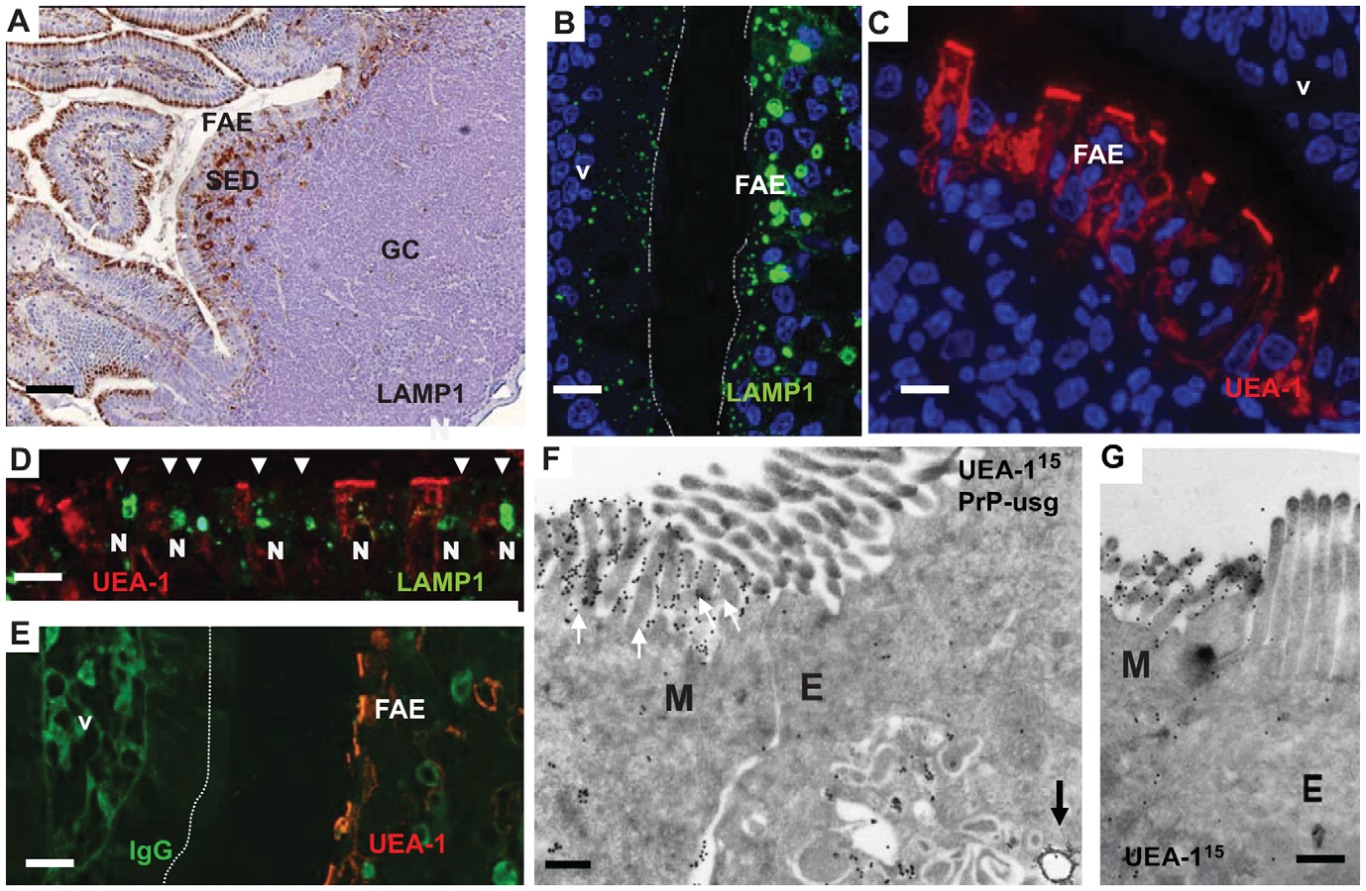
Follicle-associated epithelium (FAE) harbours enterocytes that have large apical late endosomal compartments and M cells. Peyer's patches were collected from wild-type 29/Ola mice 1 day after infection with ME7 prions. (A) Cellular organization of a Peyer's patch showing the LAMP1-positive compartments at the FAE and SED. (B) FAE enterocytes have large LAMP1-positive endosomes compared to enterocytes in the villi. (C and D) UEA-1 positive (red) M cells within the FAE. Note in (D) the large apical LAMP1+ endosomes are not present in M cells, but only in adjacent FAE enterocytes (arrowheads). N depicts the approximate location of nucleus in the epithelium (E) UEA-1 label is present only on FAE, whereas the neighbouring villus is negative. IgG (green) presents the endogenous mouse IgGs. (F and G) Cryo-immuno EM reveals that M cells in the FAE differ from enterocytes by having irregular microvilli that are UEA-1–positive (F, G). The diameter of the UEA-1–specific gold particles in (F) and (G) is 15 nm. Note in (F) the arrow points to a PrP-positive early endosomal vesicle in the UEA-1 negative enterocyte, whereas little PrP uptake is detectable in the UEA-1 positive M cell. PrP was detected with PrP-specific 6H4 monoclonal antibody directly conjugated to UltraSmall gold (PrP-usg) and visualized by silver enhancement. A larger high resolution version of Figure 1F is provided as a supplement (Figure S3). SED, subepithelial dome; GC, germinal centre; V, villus; M, M cells; E, enterocytes. Scale bars: (A) 80 µm; (B) 15 µm; (C) 10 µm; (D) 15 µm; (E) 20 µm; (F) 200 nm and (G) 200 nm.

**Figure 2:**
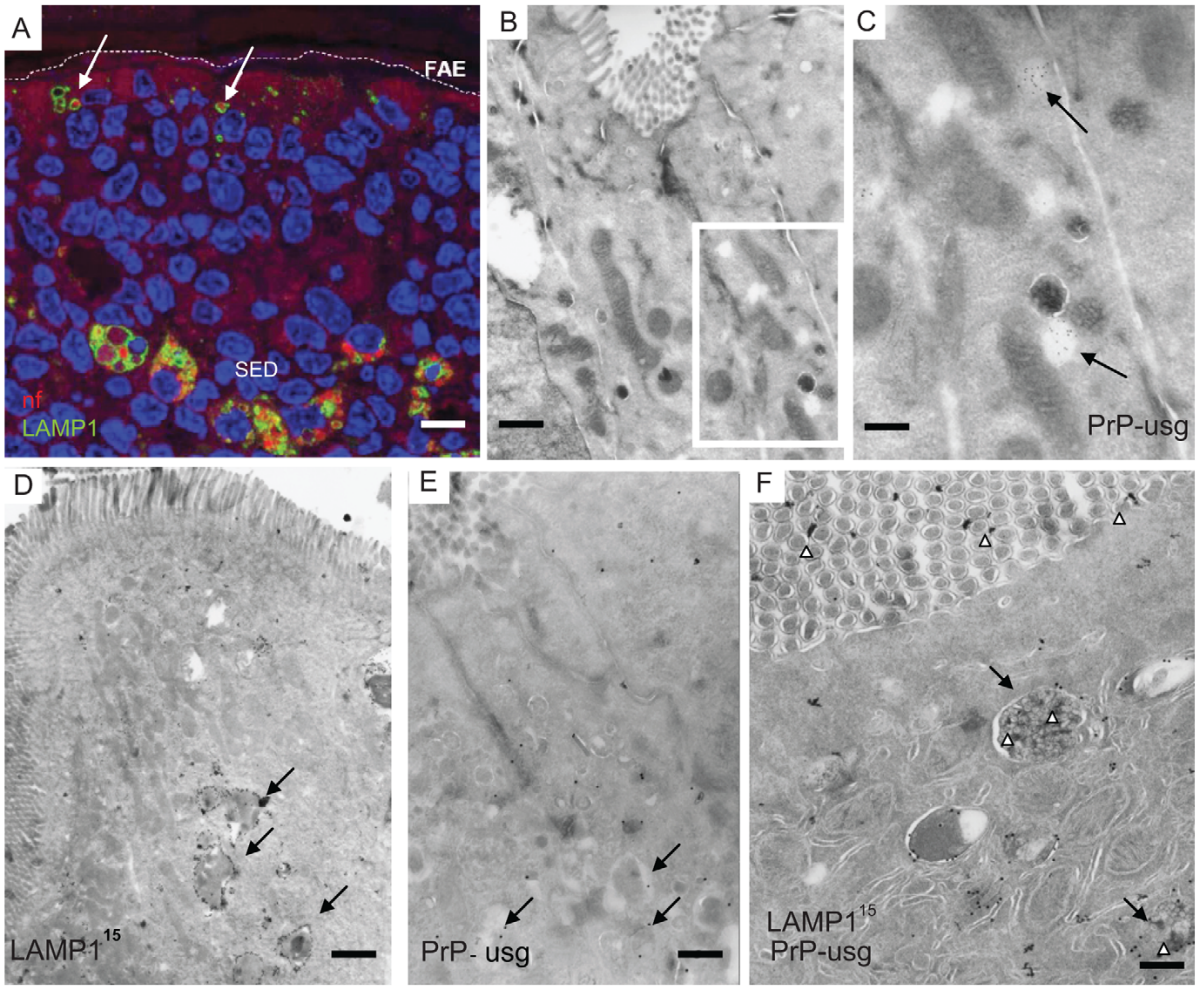
Subcellular localization of neurofilament and PrP uptake in FAE and SED. Neurofilaments (A) and PrP (B–F) were observed in wild type 129/OLA mice at 1 dpf after oral prion exposure. (A) Neurofilaments (bright red) can be detected in LAMP1-positive endosomes (green) in the FAE (white arrows) and in larger LAMP1-positive structures in macrophages in the SED. (B) PrP can occasionally be seen in small electron-lucent early endosomal structures of FAE enterocytes, shown at higher magnification in (C, arrows). (D–F) Most of the PrP signal in FAE of infected animals is found in LAMP1-positive, late endosomes of enterocytes. Late endosomes with electron-dense contents (arrows) are labelled with LAMP1 (D and F); PrP was detected with PrP-specific 6H4 monoclonal antibody directly conjugated to UltraSmall gold (PrP-usg) (E and F) visualized by silver enhancement for 5 min (E) or 1 min (F). Arrows in (E) point to PrP-usg-labelled late endosomes. (F) White arrowheads indicate PrP-specific label within LAMP1-positive multivesicular bodies and between the apical microvilli facing the intestinal lumen. Black arrows indicate LAMP1 labelling (15 nm gold) on the limiting membrane of these structures. A larger high resolution version of Figure 2F is provided as a supplement (Figure S4). Scale bars: (A) 8 µm; (B) 250 nm; (C) 125 nm; (D) 600 nm; (E) 500 nm and (F) 300 nm.

In orally infected animals, prions are suspected to enter the lymphoid and nervous systems from the intestine through the FAE-overlying follicles of the mucosa-associated lymphoid tissue [15]–[17]. Thus, we examined the FAE and subepithelial dome (SED) region of Peyer's patches in wild-type (wt) uninfected mice and in wt mice infected with ME7 or RML prions. For comparison we also examined normal villi in prion-infected and uninfected mice. Late endocytic compartments were identified using antibodies against LAMP1, a membrane protein specific for these compartments (for an overview, see Figure 1A). By immunofluorescence (IF) analysis, we found significantly larger, LAMP1-positive endosomes with a typical multivesicular body phenotype in the FAE as compared to those in neighbouring villi (Figure 1B, 1D and Figure S1). Large LAMP1-positive endosomes were also observed in macrophages in the SED region, but had different more pleiomorphic morphology and stained less intensively with LAMP1-antibody (Figure 2A, Figure S1). Large LAMP1 endosomes of FAE enterocytes were abundant, had a more regular round shape (appeared as ring-like structures in IF and histochemistry sections) and had predominantly an apical location between the nucleus and brush border. Occasionally, when macrophages invade into the FAE their LAMP1 positive endosomes retain their pleiomorphic morphology and are mostly located basolaterally between the enterocyte nucleus and the basal membrane (see Figure S1C). At EM level investigating the cellular contours of individual cells by following their plasma membranes it could be confirmed that the apical large LAMP1 positive endosomes were located in FAE enterocytes and not for example in FAE-invading macrophages (Figure S1). Similar observations were made in both prion-infected and uninfected wt mice (data not shown). These data suggest that FAE enterocytes could play an important role in transcytosing partially digested, gut-derived proteins to macrophages within the SED. The apical large LAMP1 positive endosomes of FAE enterocytes were so distinctive that their presence could be used as a useful landmark for detecting the FAE in IF and EM sections.

Epithelial M cells (or microfold cells) within FAE are considered as an entry point for many intestinal pathogens, both bacteria and viruses [18] and also proposed as portals of entry for prions [19]–[21]. We identified M cells in the FAE using *Ulex europaeus* agglutinin 1 (UEA-1) as a marker. As anticipated, UEA-1 bound strongly to the brush borders of M cells and to a lesser extent to their basolateral cell membranes and the limiting membranes of intracellular vesicles (Figure 1 C and D). The UEA-1 label was restricted to M cells in the FAE and not observed in the neighbouring villus (Figure 1E, although especially the mucus containing granules of goblet cells elsewhere in the villi were often UEA-1 positive. Occasional goblet cells could also be seen in the FAE, but were easily distinguished from the UEA-1 positive M cells by their distinct morphology. The identification of M cells in the FAE was further confirmed by using antibodies specific to the M cell markers GP-2 [34] and annexin V [35] see Figure S2. Cryo-immuno- EM revealed that UEA-1 -positive cells within the FAE had typical, short, irregular microvilli (Figure 1F and 1G, Figure S2 and S3) and often harboured lymphocytes within an intracellular pocket. Each of these observations is an established characteristic of M cells. Our analysis revealed that M cells had smaller, LAMP1-positive late endosomes and completely lacked the large late endosomes found in FAE enterocytes. Few UEA-1 positive goblet cells were seen in the FAE by EM, but were easily distinguished from M cells by their apical mucus-containing granules and pronounced ER, typical for secretory cells (see Figure S2).

### Enterocytes within the FAE take up neurofilaments from orally-acquired brain homogenate

**Figure 3:**
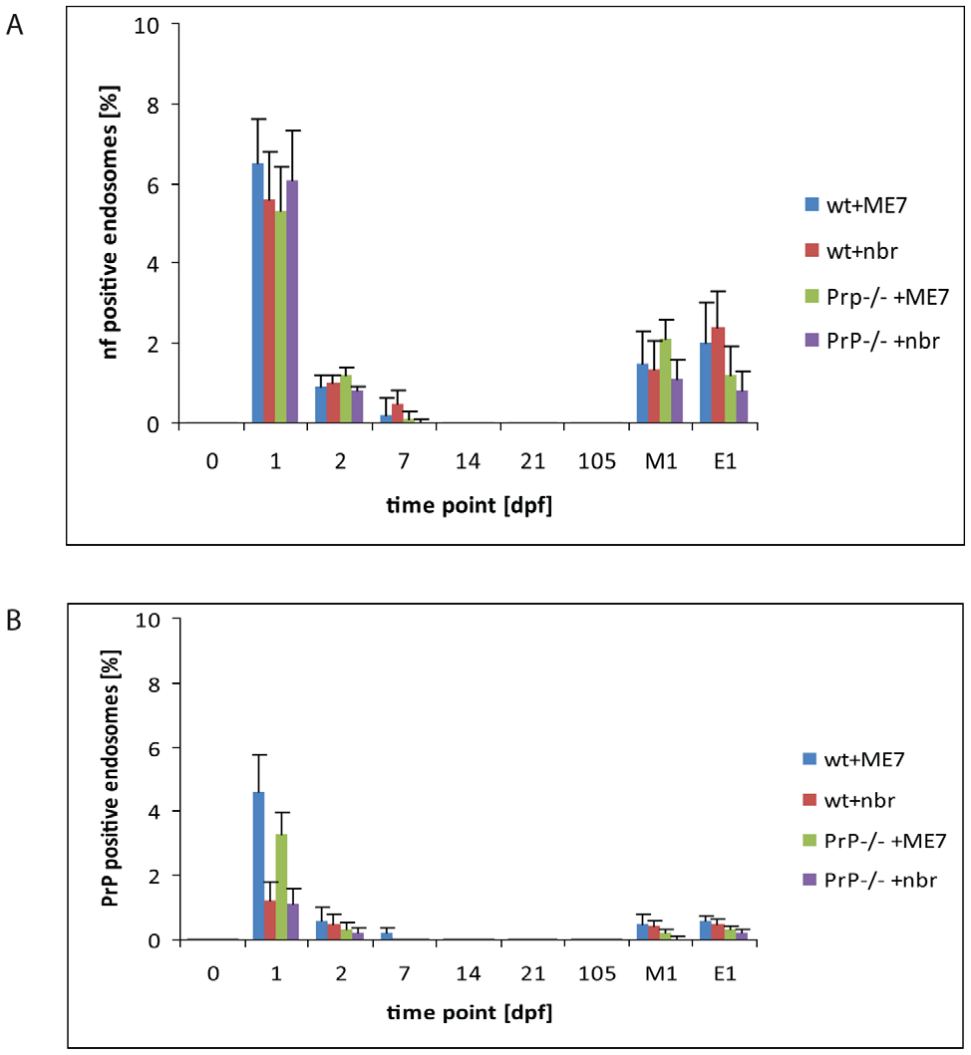
Quantification of neurofilament- and PrP-positive late endosomes. LAMP1 positive endosomes that were also positive for neurofilament (A) and PrP-specific gold particles (B) in wild-type and PrP-deficient mice following oral prion infection (in dpf, x axis). Similar quantifications of NF and PrP in wt and *Prnp* ^–/–^ mice fed normal brain homogenate (+nbr) are also shown. Mean percentages ± SD from the two most proximal Peyer's patches per mouse and two mice per group are shown for 100 randomly selected FAE enterocytes. The apical LAMP1 positive endosomes between the brush border and nucleus were counted. In this apical region there are in average between 3 to 6 LAMP1 positive endosomes per section of a cell. Analogously, 100 FAE M cells (M1) and 100 randomly selected villus enterocytes (E1) were analysed for LAMP1-positive late endosomes at 1 dpf, the time point of highest scores, are also shown. NF was examined by IF; PrP was evaluated by cryo-immunogold EM.

Next, groups of PrP-expressing wt mice and PrP-deficient (*Prnp* ^-/-^ mice) were orally exposed to prions, by feeding brain homogenates from prion-infected mice. As controls, mice were fed uninfected brain homogenate. In order to trace the fate of the brain inoculum Peyer's patches were collected at intervals after exposure, and the early cellular and subcellular localization of brain homogenate-derived neurofilaments (NF) determined by IF. Peyer's patches were also examined from age-matched mice that had not been fed brain homogenate. Both wt and *Prnp* ^-/-^ mice fed either prion-infected or uninfected brain homogenate showed positive immunolabeling for NF, whereas untreated control mice were negative for NF immunolabeling (data not shown). In Peyer's patches taken at 1 dpf, NFs were detected in FAE enterocytes within the large LAMP1-positive endosomes of mice exposed to prion and normal brain homogenate (Figure 2A, 3A). Both wt and *Prnp* ^–/–^ mice showed similar levels of NF staining in FAE enterocytes, indicating that the uptake of NF was not dependent on PrP^C^ expression (Figure 3A). Much fewer NF-positive endosomes or other NF-containing organelles were found within M cells in the FAE of Peyer's patches from the prion-exposed or control animals (Figure 3A). By immunogold-EM the prion inoculum was locally visible in the lumen of the gut (Figure 2F and Figures S3, S4 and S5) indicating that the transcytosis process of the orally introduced PrP was still going on at 1 dpf. In addition, throughout this study, no evidence was found to indicate direct uptake from the gut lumen via macrophages or dendritic cells, and few NF-positive endosomes were seen in neighbouring villi. In samples taken at later time-points (>1 dpf), the NF signals decreased in the LAMP1-positive FAE enterocytes of all mice. Together, these data suggest that the initial uptake of the brain homogenate from the gut lumen occurred mainly via enterocytes within the FAE and that the active uptake of the inoculum was still ongoing one day after feeding.

### Enterocytes within the FAE take up PrP^Sc^

To determine whether the NF immunolabeling was related to prion uptake from the gut lumen, we analysed subcellular structures of the FAE for PrP accumulation by cryo-immuno EM. No PrP-specific gold particles were found in the FAE of Peyer's patches from uninfected wt and *Prnp* ^-/-^mice (day 0; Figure 3B). In tissue obtained at 1 dpf, PrP-specific immunolabelling was detected in FAE enterocytes from all mice, regardless of their PrP^C^ expression levels. Animals fed prion-infected brain homogenate showed greater PrP labelling compared to mice fed normal brain homogenate (Figure 3B). Although the immunodetection conditions used here do not allow discrimination between cellular PrP^C^ and pathogenic PrP^Sc^, the PrP labelling in prion-exposed mice is likely due to PrP^Sc^. The PrP signal was occasionally found on electron-lucent, small early endosomal vacuoles (Figure 2B and 2C), but was most abundant within large endosomal vacuoles similar to those labelled with LAMP1 (Figure 2D–F, Figure S4 and S5). As observed for the uptake of NF, PrP accumulated in FAE enterocytes. Comparatively much lower levels were found in M cells or in the enterocytes of the neighbouring villi at 1 dpf (Figure 3B). Also similar to the NF signals, the PrP signal detected within FAE enterocytes was transient: PrP labelling was decreased in samples at 2 dpf, and undetectable by 14 dpf in both wt and *Prnp* ^–/–^ mice (Figure 3B).

In order to discriminate the “exogenous” PrP^Sc^ within the orally administered prion inoculum from “endogenous” PrP^C^ or PrP^C^ within the prion inoculum an additional set of experiments was performed. Groups of wt and *Prnp* ^-/-^ mice were fed PK-treated brain homogenate prepared from terminally Sc327 scrapie-infected hamsters. Immunoblot analysis confirmed that treatment of the inoculum with PK destroyed any PrP^C^ present within the brain homogenate leaving proteinase-resistant PrP^Sc^ (Figure S6). Peyer's patches were collected at 6 and 24 hours post infection, processed for cryo-immuno EM and sections were immunolabelled with mAb 3F4 directly conjugated to 10 nm gold particles. Since the mAb 3F4 recognizes only hamster PrP and does not label mouse PrP [36], the use of this mAb ensures any PrP detected is hamster-specific PrP^Sc^ from the Sc327-infected brain inoculum, and not PrP^C^ expressed in the host mouse. Small clusters of gold labelling, indicative of PrP^Sc^, were detected within small electron-lucent early endosomal vacuoles at 6 h postfeeding (Figure S7A and B). By 24 h after infection similar clusters of PrP^Sc^ were detected in the lumen of multivesicular bodies (Figure S7C and D). mAb 3F4-specific immunolabelling was observed predominantly in FAE enterocytes both in prion-infected wt and *Prnp* ^-/-^ mice, but was not detectable in control animals fed with PK-digested normal brain homogenate. Together, these data demonstrate that enterocytes within the FAE acquire PrP^Sc^ from the gut lumen. Furthermore, these data also confirm that PrP uptake from the gut lumen was independent of host PrP^C^ expression, and also independent of the PrP sequence of the inoculum and therefore not affected by the species barrier between hamster and mouse.

Together, these data suggest that both PrP^C^ and PrP^Sc^ cross the FAE via the endosomal system of enterocytes within the bulk flow of transcytosed material from the gut lumen. Furthermore, our data clearly show that the majority of PrP uptake from the gut lumen occurs within FAE enterocytes when compared to M cells and independently of the expression of cellular PrP^C^, since no substantial differences were observed between tissues from prion-exposed PrP-deficient and wt mice.

### Prions are delivered to macrophages within the SED

**Figure 4:**
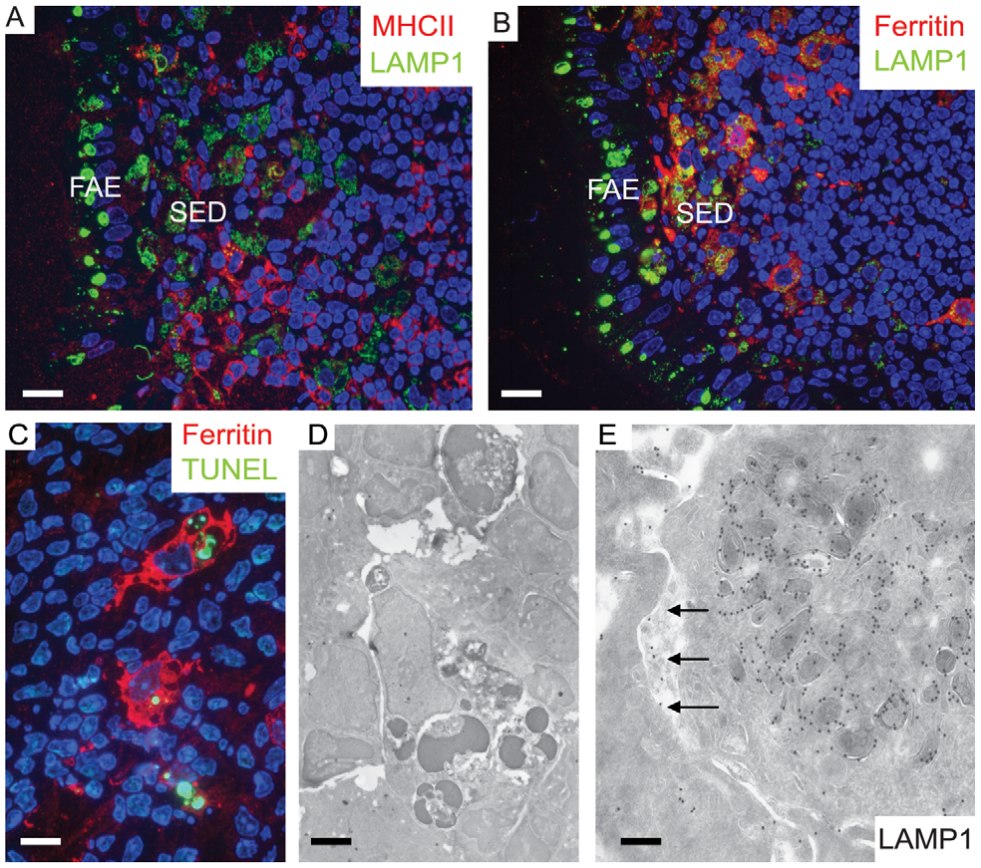
Two separate subpopulations of phagocytic monocytes in SED. In the SED a subpopulation of phagocytic monocytes with large LAMP1-positive compartments also label for ferritin, a macrophage marker. 129/Ola wild type mice were infected with ME7 prions and visualized at 1 dpf. (A) MHCII-positive dendritic cells appear to be separate populations from the cells that harbour large LAMP1 vacuoles at the SED. (B) By comparison, cells with large LAMP1 compartments also express ferritin (red). (C and D) Apoptotic lymphocytes were seen by TUNEL staining (green in C) inside ferritin-positive macrophages (red in C) and as electron-dense dark bodies in the late endosomal/lysosomal compartments (D). These typical tingible body macrophages (TBMs) were found further away from SED in the germinal centre. (E) A mononuclear phagocyte at SED appears to have exocytosed some of its LAMP1-positive endosomal compartments into the intercellular space (arrows). Scale bars: (A) and (B) 12.5 µm; (C) 8 µm; (D) 2.4 µm and (E) 200 nm.

Following transcytosis of brain material across the FAE, NFs were detected within a cluster of large LAMP1-positive organelles (Figure 2A). The SED region is known to harbour a large population of CD11c-positive cells that are often referred to as classical dendritic cells (DC), although they most likely represent a heterogeneous mixture of subpopulations of classical dendritic cells and macrophages in their different maturation forms [37], [38]. Unfortunately, CD11c-specific antibodies did not work on the aldehyde-fixed tissues used in the current study. However, most mucosal mononuclear phagocytes and all those within Peyer's patch germinal centres express CD11c indicating that this marker alone does not directly discriminate classical DC [38]. Thus, in order to discriminate between classical dendritic cells and macrophages, we used antibodies specific to major histocompability complex class II (MHC II) molecules to detect classical dendritic cells and antibodies against ferritin as a marker for macrophages (Figure 4, A and B, respectively). Macrophages express high levels of ferritin and harbour much lower levels of MHC II molecules on their surface (See Figure S8).

In 1 dpf samples, the macrophages within the SED harboured large quantities of NF (Figure 2A) which co-localized with LAMP1-positive structures. Many of these cells also appeared strongly positive for ferritin. Ferritin-positive cells that contained phagocytosed remnants of apoptotic lymphocytes, a landmark for tingible body macrophages (TBMs) were found further away from SED in the germinal centers (Figure 4C and D). In SED PrP-specific labelling was found in late endosomes of low MHC II –expressing macrophages by cryo-immuno EM (see Figure S9) indicating transcytosed prion inoculum in the SED region. Some macrophages were observed with intensely labelled intracellular LAMP1 structures, and LAMP1-positive membrane structures were also found in the surrounding extracellular space (Figure 4E). These probably secreted vesicles suggested intercellular exchange of membranous material by exocytosis and endocytosis (Figure 4E). We conclude that within the SED, PrP mostly accumulates within ferritin-positive macrophages, but not in MHC II–positive classical dendritic cells.

### PrP^Sc^ accumulation upon FDCs

**Figure 5:**
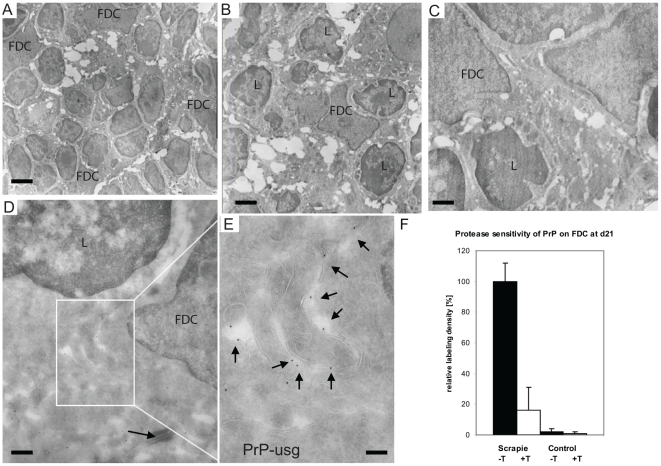
Increased PrP accumulation upon FDCs through the course of early prion disease. (A–E) In samples taken from Peyer's patches of wt mice at 21 dpf with ME7 prions, PrP accumulates in the germinal centres on the surface of mature FDCs. (A-C) Overviews show mature FDCs at the germinal centre. (D–E) In samples taken from wt mice at 21 dpf with ME7 prions, increased PrP labelling was observed on the cell surface of FDCs. Panel E shows an inset in D at higher magnification, with arrows indicating the silver enhanced mAb 6H4-conjugated UltraSmall gold PrP label (PrP-usg). The arrow in panel D indicates a desmosome. (F) Trypsin treatment of sections (white bar; scrapie +T) causes an average decrease of 84% in the amount of PrP-specific label on FDC plasma membranes of PrP-infected mice when compared to untreated sections (black bar; scrapie -T; 100%). The remaining PrP after trypsin treatment is indicative for protease-resistant disease related PrP^Sc^. Untreated (black bar, control -T) and trypsin-treated (grey bar, control +T) sections from uninfected mice show little PrP-specific label. Samples were collected at 21 dpf and the FDC plasma membrane bound PrP-specific gold was counted in untreated and trypsin treated Peyer's patch cryosections of ME7 infected mice and noninfected controls. Ten mature FDC cells in the germinal centre were randomly selected and 50 µm of plasma membrane was analysed per cell. A total membrane length of 500 µm per treatment was analysed and the result is given as a relative labelling density per membrane length ± SED. L; lymphocyte. Scale bars: (A) 5 µm; (B) 2 µm; (C) 1 µm; (D) 500 nm and (E) 200 nm.

FDCs are typically binuclear with distinct chromatin pattern characteristics, electron-lucent cytoplasm and numerous convoluted extracellular extensions surrounding lymphocytes at different stages of differentiation. These characteristics allow FDCs to be readily identified by EM [39], [40]; typical examples of FDCs engulfing B lymphocytes are shown in Figure 5A–C.

**Figure 6:**
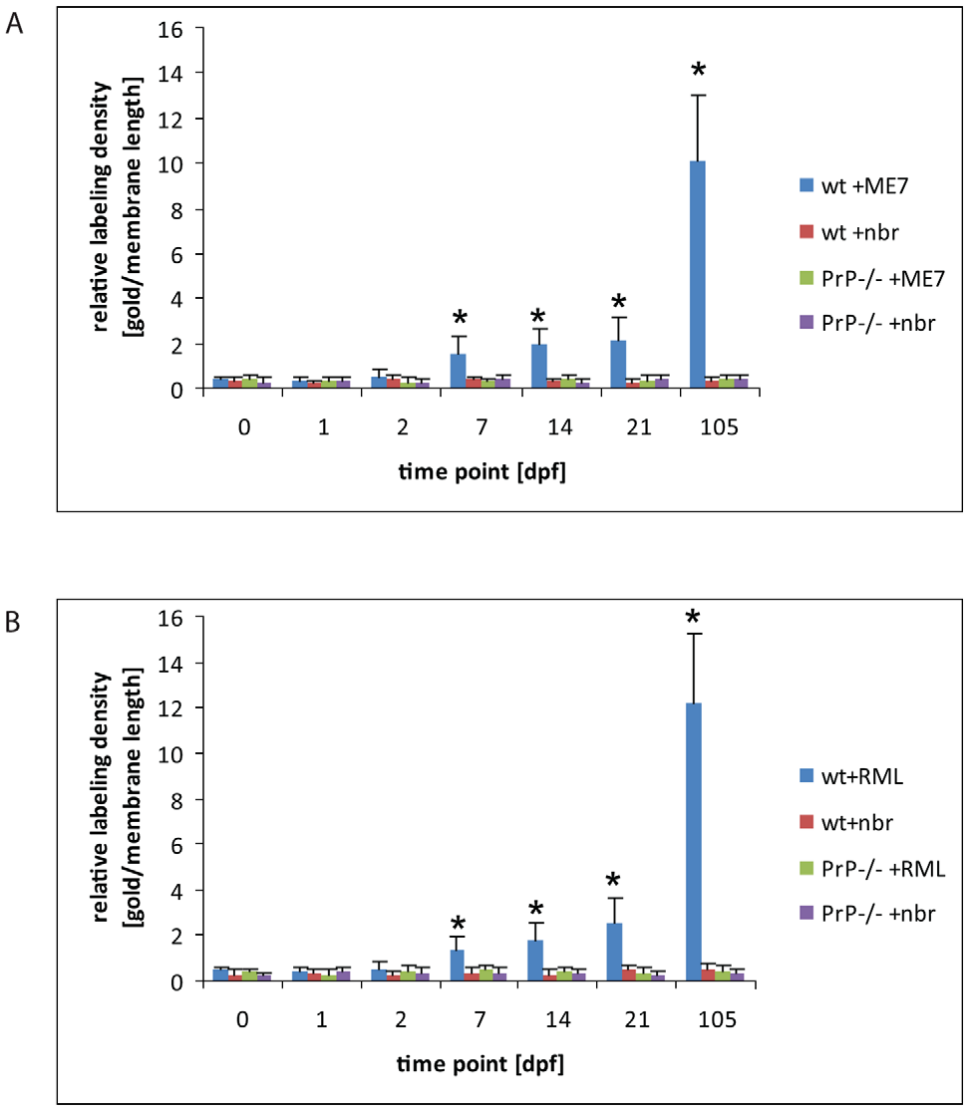
An increase of PrP label upon FDCs of wt mice only. Quantification of PrP label on the plasma membrane of FDCs indicates a clear increase of PrP label in wt mice between 2 and 7 dpf with either ME7 (A) or RML (B) prions compared to wt and PrP^-/-^ animals that were fed with normal brain homogenate (nbr) or PrP^-/-^ animals fed with ME7 or RML prions, respectively. The labelling density was counted as gold particles/membrane length (µm). The x axis indicates the number of days after oral infection at which samples were taken. A gold particle was defined as membrane associated if it was not further than 20 nm away from the membrane leaflet. The two most proximal Peyer's patches per mouse and two mice per group were analysed. Only plasma membranes of cells with typical FDC morphology in GCs (cells with lightly electron-lucent, bilobular nucleus that were in cellular contact with ≥1 non-apoptotic lymphocytes) were counted. A total membrane length of 1000 µm per sample was analysed. Asterisks indicate a statistically significant difference between wt mice infected with prions compared to other groups, p<0,05.

In wt mice, oral exposure to either ME7 or RML prions resulted in a significant increase in PrP immunolabeling on the plasma membrane of FDCs beginning at 7 dpf (Figure 6A and B), which increased and became more uniform in samples taken at 21 dpf (Figure 5D–E). At 105 dpf, locally high levels of PrP label were found on FDC plasma membranes in germinal centers of PrP-infected wt (Figure S10). The increase in PrP signal (Figure 6) was observed only in prion-infected wt mice and was restricted to the plasma membrane of FDCs These observations suggest that the increase in PrP immunolobelling on FDCs of prion-infected mice was PrP^Sc^. Initially, the increased PrP immunolabelling was often found heterogeneously on long dendritic extensions of single FDCs. Increased PrP levels were simultaneously also observed in germinal centres of mesenteric lymph nodes draining the intestines of wt mice (data not shown).

Increased PrP labelling was not observed in wt mice fed normal brain homogenate, suggesting that the labelling we found on FDCs of prion-infected mice was disease-related PrP^Sc^. No PrP immunolabelling was observed on FDC in prion-exposed *Prnp* ^–/–^ mice, indicating that the increased PrP signal was unlikely to be due to accumulation of PrP^Sc^ from the prion inoculum. No accumulation of PrP was observed in samples taken from wt spleens during the first 21 dpf (data not shown). Thus, coincident with the disappearance of PrP from FAE enterocytes, PrP accumulated on the plasma membrane of FDCs within Peyer's patches and mesenteric lymph nodes in a PrP^C^-dependent fashion. Together, these data imply the first site of PrP^Sc^ conversion and replication following oral exposure.

To characterise the PrP on FDCs, and to investigate whether protease-resistant PrP^Sc^ had formed, sections were additionally treated with trypsin before immunolabeling with R2. This antibody recognises an epitope of PrP containing a trypsin cleavage site [41], and as seen previously for sections of mouse brain infected with RML prions [42], little R2 labelling of PrP^C^ or PrP^Sc^ remained on FDC plasma membranes after the treatment, either at 21 days (Figure 5F) or 105 dpf (Figure S10).

### Enhanced tingible body macrophage activity within Peyer's patches from prion-infected mice

**Figure 7:**
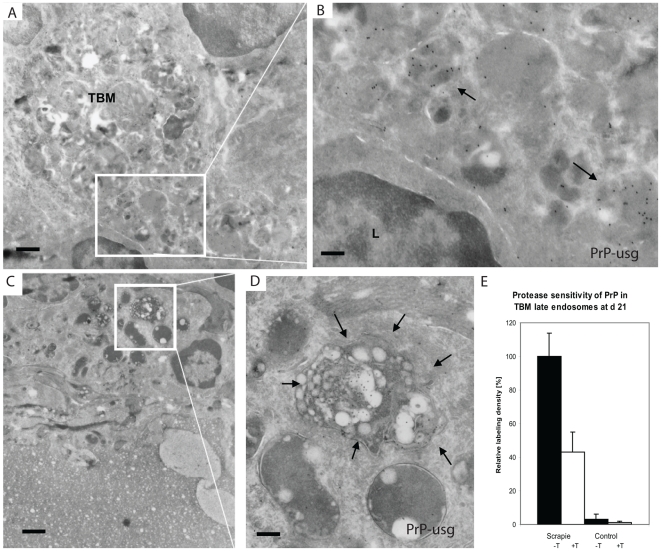
PrP positive endosomes in the germinal centre of Peyer's patch of wt mouse exposed to ME7 prions. Silver enhanced cryo-immuno EM labeling of PrP with mAb 6H4 conjugated to UltraSmall gold (PrP-usg) shows increased numbers of PrP-positive late endosomes and lysosomes of tingible body macrophages (TBMs) in germinal centres of wt mice at 21 dpf. Areas framed by white lines in panels A and C are shown at higher magnification in panels B and D, respectively. Arrows in B and D point to PrP-positive, late endosomes/lysosomes. L; lymphocyte. (E) After trypsin treatment an average of 48% of the PrP-specific label (white bar; scrapie +T) remains in the lumen of late endosomes/lysosomes of PrP-infected mice when compared to untreated PrP-infected mice (black bar; scrapie -T; 100%). The remaining PrP after trypsin treatment is indicative for protease-resistant disease related PrP^Sc^. Untreated (black bar, control -T) and trypsin-treated (grey bar, control +T) uninfected mice show little late endosome/lysosome associated PrP-specific label. Samples were collected at 21 dpf and the PrP-specific gold was counted in the lumen of late endosomes/lysosomes of tingle body macrophages in germinal centres of untreated and trypsin treated cryosections of Peyer's patch from ME7 infected mice and noninfected controls. A total of 100 late endosomes/lysosomes per treatment were analysed and the result is given as a relative labeling density per area ± SED. Scale bars: (A) and (C): 1 µm; (B) 300 nm and (D) 250 nm.

Further IF and cryo-immuno EM of cells in the vicinity of FDCs revealed PrP within LAMP1-positive, late endocytic compartments in cells that had all the morphological landmarks of tingible body macrophages (TBMs) (Figure 4C and D; Figure 7; Figure S10). TBMs are a subset of large mononuclear phagocytes that reside in germinal centers of secondary lymphoid tissues. TBM contain many phagocytised apoptotic cells in various states of degradation (referred as tingible bodies). The detection of PrP within these compartments implied that these cells were most probably scavenging PrP. The number of TBM cell profiles positive for PrP increased during the course of prion infection, suggesting enhanced TBM activity in the Peyer's patches of infected wt mice (Figure S11). Compared to FDCs at the same time points, a higher portion of the PrP labelling of R2 antibody in the late endosomes / lysosomes of TBMs was unaffected after trypsin treatment (Figure 7E and S10F), suggesting that the early PrP labelling on FDC plasma membranes may be protease-sensitive PrP^Sc^, but that PrP^Sc^ targeted for degradation may have acquired a degree of protease resistance. Again, tissues from uninfected mice and prion-infected *Prnp* ^-/-^ mice did not show PrP in TBMs (data not shown), which argues that this increase is specific to the propagation of PrP^Sc^.

### Early detection of prion infection within the enteric nervous system

**Figure 8:**
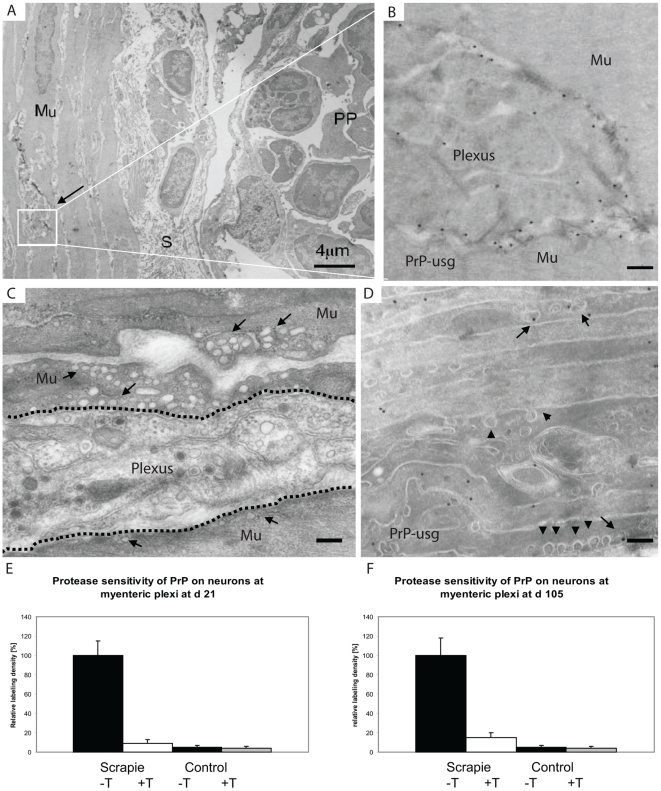
First PrP observed in myenteric plexi. PrP labelling is observed in myenteric plexi in samples from wt mice taken at 21 dpf. (A) Overview of the edge of a Peyer's patch (PP), submucosa (S) and the underlying muscle layer (Mu). The arrow points to the myenteric plexus. (B) Higher magnification view of the plexus (boxed area in A) shows a high density of silver enhanced PrP label (mAb 6H4 conjugated to UltraSmall gold (PrP-usg)) on the plasma membrane of myenteric neurons. (C and D) Micrographs show that the smooth muscle cells surrounding the plexi have numerous electron lucent caveosomes on their surface (arrows in C indicate caveosome-rich areas, arrowheads in D indicate individual caveosomes), some of which are PrP-positive (arrows in D). Dotted line in C depicts the border between the plexus and muscle layer. (E and F) After trypsin treatment an average of 9% at 21 dpf (E) and 15% at 105 dpf (F) of the PrP-specific label (white bar, scrapie +T) remains on the surface of neurons at myenteric plexi of PrP-infected mice when compared to untreated PrP-infected mice (black bar; scrapie -T ; 100%). Untreated (black bar,control -T) and trypsin-treated (grey bar, control +T) uninfected mice show little myenteric plexi associated PrP-specific label. The PrP-specific gold was counted on the surface of neurons at the myenteric plexi of untreated and trypsin treated cryosections of Peyer's patch from ME7 infected mice and noninfected controls. A total of 10 myenteric plexi per treatment were analysed and the result is given as a relative labelling density per area ± SED. Scale bars: (A) 4 µm; (B) 300 nm; (C) 250 nm and (D) 200 nm.

In samples from prion-infected wt mice obtained at 21 dpf, the first signs of PrP accumulation within myenteric (Auerbach's) plexi were observed between the inner circular and outer longitudinal layers of the muscularis in regions close to Peyer's patches (Figure 8A and B). In samples from prion-infected, wt mice obtained at 105 dpf, almost all the plexi closely associated with Peyer's patches had strong accumulations of PrP (data not shown). Congruent with observations above, increased PrP was only detected within the enteric nervous systems of wt mice exposed to prion-infected brain homogenate. We have previously shown that caveosomes are involved in intracellular PrP^C^ trafficking in cultured CHO cells [43]. We found no evidence for a role of caveosomes in PrP trafficking *in vivo* within the stroma of Peyer's patches. However, smooth muscle cells neighbouring infected plexi appeared rich in caveosomes (Figure 8C), which often contained PrP (Figure 8D), implying a potential mechanism through which PrP may be disseminated within the muscle layer of the intestine. After trypsin treatment of cryosections of prion-infected wt animals, approximately 9% of PrP label remained on plasma membranes of neurons at myenteric plexi of sections collected at 21 dpf, and increased to 15% for sections obtained at 105 dpf, indicating the presence of protease-resistant PrP^Sc^ on enteric nerves at relatively early phases of prion infection (Figure 8E and F).

### A33 links the FAE to FDCs within lymphoid follicles

**Figure 9:**
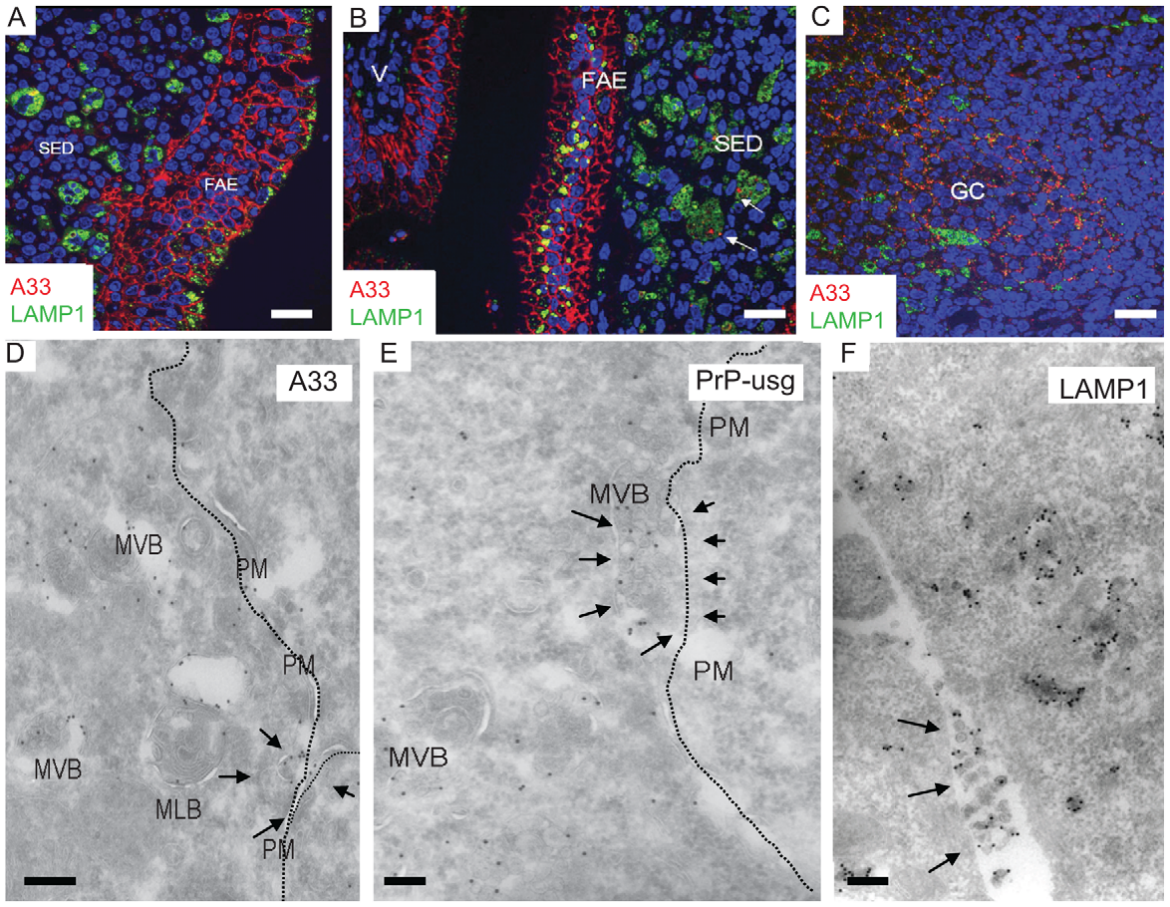
The endothelial marker A33 is found in FAE and SED of Peyer's patches from wt mice. (A) A33 (red) labels basolateral plasma membranes of FAE enterocytes. (B and C) Some A33-positive membrane components can be found in association with LAMP1-positive (green) subcellular compartments on (B) dendritic cells/macrophages in the subepithelial dome (SED; arrows) as well as (C) on the cell surfaces of the FDC network on germinal centres (GC). (D–F) Some multivesicular bodies of FAE seem to fuse with the plasma membrane (arrows in D and E; PM; dotted line), releasing their intravacuolar membranes as exosomes into the extracellular space (arrows in F). For these images 129/Ola wt mice were infected and analysed at 7 dpf. V; villus. Labeled antigens: A33 (D); PrP (silver enhanced mAb 6H4 conjugated to UltraSmall gold, PrP-usg) (E) and LAMP1 (F). Scale bars: (A–C) 20 µm; (D–E) 200 nm; and (F) 250 nm.

The definitive epithelial marker A33 (*Gpa33*; [44]) is expressed at high levels on the basolateral plasma membrane of the FAE and villous enterocytes (Figure 9A). Although *Gpa33* is not expressed by macrophages and classical dendritic cells (See Figure S12), cryoimmuno EM revealed the presence of A33 protein within the endosomes of SED macrophages, indicating delivery of A33-positive membranes from enterocytes to macrophages (Figure 9B). Careful analysis revealed A33-positive vesicles within the germinal centres of Peyer's patches (Figure 9C) from each mouse group. In contrast, no A33 immunostaining was detected in Peyer's patches from A33-deficient control mice, confirming the specificity of the A33-specific antibody (Figure S13).

Weak A33 immunolabeling was also observed within the germinal centrers of mesenteric lymph nodes, whereas those in the spleen were negative (data not shown). The presence of small amounts of A33 antigen within the germinal centres of mesenteric lymph nodes most likely represents membrane trafficking from the gut epithelium to the B cell follicles as described [45]. Within the germinal centres, A33 was present on the surface of FDCs and often in close proximity to TBMs. It is therefore tempting to speculate that the scavenging macrophages may be carrying epithelial membrane components (including PrP^Sc^ on A33-positive membranes) from the FAE to the germinal centre, where they are deposited on the surface of the FDCs. Our EM data (Figure 9) suggest that LAMP1-positive, late endocytic multivesicular bodies of FAE enterocytes fuse with the plasma membrane [46], releasing their intravesicular membrane contents as exosomes in the extracellular space, where they may be phagocytosed within the SED by macrophages and further spread in a “taste and spit” -manner of repetitive endo- and exocytosis events by macrophages. Consistant with this idea, exosome-like membrane structures were shown to be positive for A33, PrP, and LAMP1 (Figure 9D–F, respectively). Thus, together these data provide further evidence for the association of FAE enterocyte-derived antigens such as A33 upon the surface of FDCs within Peyer's patches and mesenteric lymph nodes.

## Discussion

Over the last few years, it has been noted that several amyloid or protein-misfolding diseases are characterized by prion-like propagation mechanisms [47], [48]. The main difference that distinguishes the prion diseases from other amyloid or protein-misfolding diseases is the fact that prions can be transmitted from one animal to another as truly infectious diseases. In many cases, this transmission occurs via the oral route, emphasizing the relevance of the present study. Here, we analyzed the initial uptake of PrP from the intestinal lumen via enterocytes of the FAE. While this initial uptake proved to be independent of the expression of cellular PrP^C^, the subsequent propagation and disease progression required an endogenous supply of PrP^C^.

The precise cellular mechanism through which prions are acquired from the gut lumen and transferred to FDCs within Peyer's patches is not known, although it has been shown that the accumulation of prions upon FDCs within Peyer's patches is critical for their efficient spread to the enteric nervous system [9], [12], [14]. Many other aspects of prion invasion have remained hypothetical, especially due to the lack of physiologically relevant *in-vivo* studies performed at the subcellular level. Prion detection during the early stages of oral infection has also been a problem. In previous immuno EM studies, PrP^Sc^ was first detected 70 days after oral exposure and only in denatured samples [11], [22].

We were able to investigate earlier stages after oral inoculation, by using adaptations of high-resolution methods developed in previous studies [42], [49]. The GALT are filled with endogenous immunoglobulins, which limit the use of mouse antibodies for immunodetection. However, many PrP-specific monoclonal antibodies (e.g., mAb 6H4 [50] and mAb R2 [51]) were raised in *Prnp* ^–/–^ mice. To avoid interference of endogenous mouse immunoglobulins and to permit better penetration in the tissue analysed, we collaborated with Aurion (Wageningen, The Netherlands) to conjugate mAb 6H4 and Fab fragments mAb R2 to UltraSmall gold particles. The PrP binding sites were visualised by silver enhancement, which enabled us to detect directly by EM the binding sites of the primary PrP antibody. Unconjugated R2 Fab fragments and UltraSmall gold-labelled Fab fragments of another PrP antibody have previously been used for IF and cryo-immuno EM on normal and prion-infected mouse brain, and R2 has been shown to recognize both PrP^C^ and PrP^Sc^ on undenatured tissue sections [42], [51]. By applying these novel PrP antibody derivates, we gained higher PrP detection levels and were able to follow PrP trafficking in nondenatured GALT as early as 1 dpf, as inoculated PrP, and 7 dpf, as replicating PrP on the surface of FDCs.

**Figure 10:**
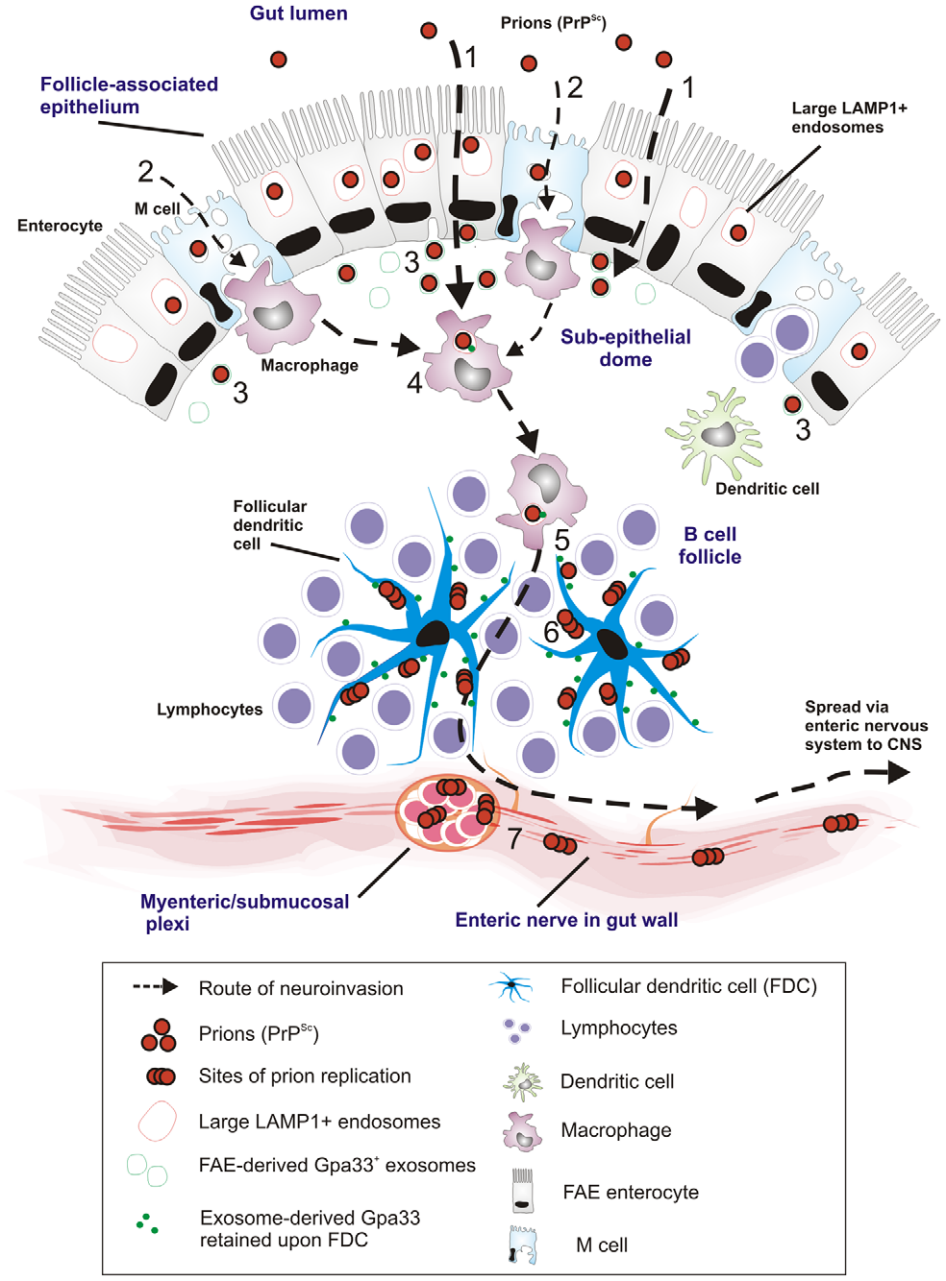
Proposed model for the for prion neuroinvasion from gut lumen via Peyer's patches to enteric nervous system. (1) Data in the current study suggest that prion uptake from gut lumen occurs predominantly via large LAMP1 positive endosomes of FAE enterocytes with (2) much lower levels via M cells. (3) FAE enterocytes release prions to SED with Gpa33+ exosomes. (4) SED macrophages uptake and release Gpa33+ exosomes and prions to their environment. (5) Prions and Gpa33+ exosomes spread to the germinal centers by TBMs (6) Prions and FAE-derived Gpa33+ exosomes accumulate on surface of FDCs in the germinal centers. Prions start to replicate on the surface of FDCs. (7) Prions replicate and spread to CNS via enteric nerves close to Peyer's patches.

The results of our time-course studies, employing IF and cryo-immuno EM with two distinct *in-vivo* mouse models, revealed the cellular and intracellular sites of prion trafficking after oral prion infection. Figure 10 represents a proposed model for prion neuroinvasion from gut lumen via Peyer's patches to enteric nervous system based on the results of the present study. In all gut samples examined, we found PrP accumulation in large, late endocytic multivesicular bodies of FAE enterocytes. Although PrP was also observed within M cells, this was at much lower levels than that within FAE enterocytes. Furthermore, our data indicated that PrP transcytosis across the FAE was independent of cellular PrP^C^ expression. In prion-infected wt mice, we subsequently found PrP in late endosomes of SED macrophages followed by a gradual increase upon the surface of FDCs within germinal centres. Relatively large amounts of PrP could also be found in late endosomes of TBMs in germinal centres. Subsequently, beginning at 21 dpf, and following accumulation upon FDCs, increased levels of PrP were observed on the surface of neurons at submucosal and myenteric plexi. At 105 dpf (approximately one third through the incubation period for 129/Ola mice orally exposed to ME7 scrapie prions) PrP-immunolabelling on neurons had increased.

Our observations of increasing PrP on FDCs are consistent with previous reports. However, our data did not support the prevailing view concerning the initial uptake and trafficking of orally administered prions. In the gut, the FAE-overlying follicles of the mucosa-associated lymphoid tissue are key players in initiating mucosal immune responses and are strongly suspected to be the major entry site from the intestine into the lymphoid and nervous systems in animals orally infected with prions [22]. Within the FAE specifically, an *in-vitro* study suggested that M cells were the main target and gatekeepers for prion invasion [18], [19], [21]. In contrast, data from our *in-vivo* study suggest that M cells are not the primary target for prion entry through the FAE. Instead, most of the PrP was found in the endosomes of enterocytes within the FAE. These appear to be specialized enterocytes with enlarged late endosomal compartments compared to the “normal” enterocytes in the villi.

Data in the current study appear to differ significantly between those data in the study by Foster et al. [21] where PrP was mainly detected in association with Peyer's patch and cecal patch M cells. The reasons for this apparent discrepancy are uncertain. In the current study mice were fed doses of scrapie prions that require amplification in the GALT prior to neuroinvasion [9], [25]. The use of such physiologically-relevant doses is important as direct neuroinvasion can occur following exposure to higher doses. In the Foster study [21] a substantially higher dose of prions was delivered directly into the stomach of recipient mice than that used in the current study. Due to the high concentration of prions within the intestine it is plausible that other cell populations were principally involved in acquiring prions.

We found indirect evidence that the FAE enterocytes may exocytose the intravacuolar contents (including endocytosed PrP from the gut lumen) of their late endosomes into the extracellular space of the SED. These exosome-like structures (for a review, see [52]) are LAMP1- and A33-positive and may be part of normal machinery [45] for antigen presentation to the immune system, since we found traces of the epithelial marker A33 within macrophages and upon FDCs. The concept of exosomes as membrane carriers was originally described in [53]. A33-related exosome secretion from intestinal epithelium has been described in general [54] and specifically related to prion transmission upon oral challenge [55]. However, it is difficult to find direct evidence for this highly dynamic process at the ultrastructural level in *in-vivo* models.

Genetic ablation of PrP abrogates susceptibility of mice to prion diseases [4]. For PrP trancytosis through the FAE, PrP^C^ is not required, since transcytosis also occurs in PrP-deficient animals. Therefore, we conclude that PrP is likely to be transcytosed across the FAE in a non-specific manner within the bulk flow of other lumen-derived, digested, and endocytosed material. After transcytosis through the FAE, the migrating CD11c-positive cells (generally considered to be classical dendritic cells within the SED) are thought to endocytose and transfer the prions to germinal centres where prion replication may occur [25]. From there, by as-of-yet unknown cellular mechanisms, the prions are able to infect the peripheral enteric nervous system and gain further access to the main pathological target of prion diseases, the central nervous system. In macrophages of the SED, we found PrP in LAMP1-positive compartments of ferritin-positive cells and to a lesser extent in MHC II-positive cells (Figure S9). These observations suggest that PrP trafficking through the SED occurs in endosomal compartments of more macrophage-like mononuclear phagocytic cells rather than classical dendritic cells. Furthermore, in the SED, PrP was only detected in endosomal compartments of these macrophages and not on the cell surface. Transient depletion of CD11c-positive cells before oral exposure to prions has been shown to markedly delay the course of neuroinvasion [25]. When we retrospectively analysed the Peyer's patches of these CD11c-positive-cell-depleted mice (diphtheria toxin–treated, CD11c-diphtheria toxin receptor-transgenic mice), we found that the ferritin-positive macrophages within the SED were likewise transiently depleted (unpublished data). Interestingly, protease-resistant PrP has been reported to be transcytosed in a complex together with ferritin through intestinal epithelial cells [23], but the relevance of this finding for the ferritin-positive macrophages that are active in PrP endocytosis remains unclear.

The subsequent accumulation of PrP upon the surface of FDCs was only observed in Peyer's patches from orally exposed wt mice, and increased throughout the duration of the experiment. These PrP^C^-expressing, nonmigratory cells have a limited endosomal/phagocytic apparatus and are specialized to present native immuno complexes on their surface. These observations strongly suggest conversion and replication of PrP^Sc^ *de novo* upon the surfaces of FDCs and not merely the accumulation of inoculated PrP^Sc^.

Taken together, these observations implicate two cellular compartments with distinct roles during the initial stages of orally-acquired prion disease: (1) endosomal compartments active in transient transcytosis and/or degradation/storage of PrP^Sc^ (LAMP1-positive endosomes in FAE enterocytes, M cells, villous enterocytes, SED macrophages, and germinal centre TBMs, and caveosomes in submucosal smooth muscle cells) and (2) the cell surface of FDCs and enteric neurons, where prion replication most likely occurs. Importantly, no increase of PrP surface labelling was observed on macrophages in the course of the early infection, even if they had high labelling densities in their endosomal compartments. Our data suggest uptake into the endosomal compartments occurs independently of cellular PrP^C^ expression, whereas PrP accumulation upon FDCs and enteric neurons is critically dependent upon the expression of PrP^C^. Further, we were able to confirm the relevance of our results by using three different mouse infection models, with *Prnp* ^-/-^ mice resistant to disease as controls.

In our previous cryoimmuno EM studies, on FVB mouse hippocampus infected with RML prions, the PrP^C^ levels were also specifically examined and were not found to increase as a result of prion disease. Increases in R2 labelling could thus be attributed to the formation of PrP^Sc^. This labelling was also mainly on plasma membranes and on early endocytic or recycling vesicles rather than late endosomal compartments [42].

A high proportion of the R2 labelling in RML prion-infected FVB hippocampus was found to be trypsin sensitive [42], as was the case with the labelling on FDCs and enteric neurons in the present study. These results are consistent with reports that protease-sensitive forms of PrP^Sc^ predominate in some types of prion disease [56], [57]. Prions in the oral inoculum probably included protease-resistant forms that were able to withstand the general proteolytic degradation that occurs in the intestinal lumen and be processed by the endosomal machinery of FAE enterocytes without losing their infectivity.

We observed a spread of PrP^Sc^ to secondary lymphoid tissues. This may be due to several different mechanisms. First, PrP^Sc^ may spread by cell-cell contacts within the Peyer's patches and via lymphatics to the mesenteric lymph nodes. Additionally, the local concentration of the prion inoculum appears to have an impact on the speed of disease progression, which could explain why PrP accumulates first in Peyer's patches, where the enlarged endocytic capacity of FAE enterocytes sufficiently loads the locally dense population of SED macrophages.

We cannot exclude the possibility that the intestinal routing of prions after oral exposure may vary depending on the combination of prion and host strain. To address this issue in the current study, we used two different mouse-passage prion isolates that have been shown to have distinct cellular requirements for replication in lymphoid tissues [2], [27] and the hamster-passaged Sc237 scrapie strain. Our analysis showed consistent data from each of these distinct prion agents. However, there are examples of other prion strain and host combinations that do not appear to require accumulation and amplification in the GALT prior to neuroinvasion, such as BSE in cattle [58], some strains of TME [59], CWD in some cervid species [60] and atypical scrapie [61]. Of course, in each of these examples, the prions must still be transcytosed across the FAE prior to establishing infection within enteric nerves in the submucosa.

At 105 dpf (the latest time point we examined), the increased PrP labelling remained restricted to germinal centres and their neighbouring enteric plexi. For clarity, at 105 dpf, the animals infected with ME7 did not show any clinical symptoms of prion disease. The onset of clinical signs were observed at approximately 272 dpf at the dose we used in our studies. PrP^Sc^ has been reported to be found in the FAE of perorally challenged hamsters at 60 days [6] or 69 days [8] after intestinal infection with 263K scrapie, and it has been suggested that the FAE might serve as a site for prion release from the host some time after oral infection. In our study, we did not see a spread of PrP^Sc^ back to the FAE. 263K scrapie is considered to be a highly neurotropic prion strain, whereas ME7 is considered more lymphotropic. One could argue that during 263K scrapie infection, PrP^Sc^ returns to the gut epithelium via infected enteric nerves which presumably infiltrate the FAE. The same nerves may have been infected at the time of prion exposure. This does not appear to occur in mice, or if it does, it happens at later stages in the incubation period.

Together, these data suggest that uptake via large late endosomal compartments of FAE enterocytes represents a novel potential M cell-independent mechanism through which prions are acquired from the gut lumen. While these data do not exclude a role for M cells or villous enterocytes in the initial uptake of prions from the gut lumen, much lower levels of PrP were detected within them when compared to FAE enterocytes. Our data show that the transcytosis of prions to the germinal centres of Peyer's patches is PrP^C^-independent as it occurs also in PrP^C^-deficient animals. In contrast, PrP^C^ expression is required for the observed high labelling densities on plasma membranes of FDCs and enteric neurons. Indeed, our data suggest that FDCs within Peyer's patches are the first site of prion conversion and replication after oral exposure. These findings provide insight into the subcellular localisation and trafficking of prions, which might provide suitable targets to arrest oral prion infection. Furthermore, these data identify a novel, previously unrecognised, enterocyte-dependent route of prion uptake and transfer from the gut lumen that may have an important influence on susceptibility to oral prion infection.

## Materials and Methods

### Mice and oral prion exposure

*Prnp* ^-/-^ mice were bred and maintained on a 129/Ola background [33]. Age- and sex-matched 129/Ola mice were used as wt controls. For oral infection with ME7 prions, both wt and *Prnp* ^-/-^ (*n* = 12 for each group) were fed individual food pellets doused with 50 µl of a 1% (wt/vol) brain homogenate prepared from wt mice terminally affected with ME7 scrapie prions. Food pellets doused with 50 µl of a 1% (wt/vol) brain homogenate prepared from uninfected mice (“normal brain homogenate”) were used as a control. These experiments were approved by the Roslin Institute's Protocols and Ethics Committee and carried out according to the strict regulations of the UK Home Office ‘Animals (scientific procedures) Act 1986’.

For oral infection with RML prions, wt FVB and *Prnp* ^-/-^ mice (*n* = 10 for each group) were infected by gavage with 100 µl of 1% brain homogenate from wt mice infected with mouse-adapted RML scrapie prions. An additional group of 6 wt and 6 *Prnp* ^-/-^mice was infected with 1% brain homogenate from Syrian hamsters infected with hamster-adapted Sc237 scrapie prions. As respective controls, 100 µl of 1% brain homogenates prepared from uninfected wt mice or Syrian hamsters was administered by gavage as well. The brain homogenates from infected and uninfected Syrian hamsters were treated with proteinase K before being administered to the mice (See legend of Figure S6 for more details). The ME7 and RML samples were used without proteinase treatment. Use of these mice was according to the Public Health Services/National Institutes of Health Guide for the Care and Use of Laboratory animals. A33-deficient mice were created as described [62].

### Tissue collection, fixation and cryo-sectioning

Mice were culled at 0, 1, 2, 7, 14, 21 and 105 dpf, and Peyer's patches, mesenteric lymph nodes, and spleens were collected and prepared for IF and cryoimmuno EM. Briefly, tissue samples were dissected from animals and immersion-fixed in a solution containing 2% paraformaldeyde and 0.2% glutaraldehyde in PHEM-buffer (25 mM HEPES, 10 mM EGTA, 60 mM PIPES, 2 mM MgCl_2_; pH 7.2). Fixed tissues were embedded in gelatin, infused in sucrose, and frozen in liquid nitrogen [63]. Frozen samples were cut on a cryo-ultramicrotome as semithin sections for IF (200 nm, -100°C) or as ultrathin sections for cryoimmuno EM (70 nm, -120°C). Sections were picked up with sucrose for IF or with a mixture of methylcellulose/sucrose for cryo-immuno EM.

### Reagents and antibodies

M cells were identified with biotinylated *Ulex europaeus* (UEA-1) lectin (L8262; Sigma). Bound lectin was detected with polyclonal rabbit anti-biotin (Rockland). The following primary antibodies were used for immunolocalisation by IF and/or cryo-immuno EM: monoclonal rat anti-LAMP1 (1D4B; BD Biosciences Pharmingen); rabbit polyclonals MHCII (JV2; generous gift from Dr. Hidde Ploegh); anti-neurofilament 200 (Sigma); anti-ferritin (F5012; Sigma); goat polyclonal anti-A33 (AF2756; R&D Systems); rabbit polyclonal anti-annexin V (Ab14196; Abcam); rat monoclonal anti-GP2/glycoprotein 2 (D277-3; MBL); monoclonal anti-PrP 6H4 [49] R2 [50] and polyclonal 1B3 [64]. To detect hamster PrP biotinylated mAb 3F4 (SIG-39640 Covance Signet Antibodies) [36] directly conjugated to Streptavidin Gold Nanoparticles (Nanocs) was used. The following secondary bridging antibodies were used for cryo-immuno EM when rat or goat primary antibodies were applied: polyclonal rabbit anti-rat and rabbit anti-goat (DAKO), respectively.

### Immunofluorescence analysis

For IF microscopy, semithin cryosections on glass slides were labelled with primary antibody, followed by species-specific secondary antibodies coupled to Alexa Fluor 488 (green) or Texas red dyes (Invitrogen, Paisley, UK). Sections were mounted in fluorescent mounting medium (DakoCytomation) and examined using a Zeiss LSM5 confocal microscope (Zeiss, Welwyn Garden City, UK). Simultaneous Dapi staining was applied to visualise the nuclei and the cellular organisation of the tissue. To prevent possible false-positive signals caused by autofluorescence, sections were treated with 1% sodium borate for 5 min. A TUNEL assay was performed using the fluorescein-conjugated *in situ* Cell death Detection kit (Roche Applied Science) according to the manufacturer's instructions.

### Cryo-immunogold EM

For cryoimmunogold EM, PrP-specific 6H4 [49] and Fab-fragments of R2 antibodies [50] were conjugated to UltraSmall gold particles (0.8 nm; Aurion, Wageningen, The Netherlands) to allow increased penetration into the cryosections and circumvent labeling artefacts caused by cross-reaction with endogenous immunoglobulins in the tissue. Labeling reactions were performed under native conditions; no antigen retrieval method was applied. The R-GENT SE-EM silver enhancement kit from Aurion was used according to the manufacturer's instructions. For other immunogold-labelling experiments, the primary antibody, or the bridging antibody, was detected via the standard protein A–gold method [63]. For double labelling, conjugates of PrP-specific antibody and UltraSmall gold were applied and briefly silver enhanced, prior to incubation with the second primary antibody that was subsequently detected by protein A–gold.

The immunolabeling of sections was done as described previously [63]. In brief, after blocking with 1% cold fish gelatin and 1% bovine serum albumin for 15 min, sections were incubated with primary antibody for 60 min, washed, and bridging rabbit antibodies were applied for 30 min when necessary. Sections were then incubated with protein A-gold (15 nm) for 20 min. The specificities of the antibodies were controlled by omission of the primary antibody. Labeled sections were viewed with a Philips CM10 electron microscope (FEI Company, Eindhoven, The Netherlands) at 80 kV.

### Quantification

The quantification of the distribution of gold particles was done according to routine stereological methods in double-blind fashion. Data are presented as means ± SD. Data were analysed using a T-test and differences were considered significant when *p*<0.05.

## Supporting Information

Figure S1 **FAE enterocytes have larger late endosomes than enterocytes in the neigbouring villi or M cells in FAE.** (A) and (B) show IF images of FAE double labelled with epithelium-specific A33 (red) and late endosomal marker LAMP1 (green). Nuclei are labelled with Dapi (blue). The large FAE endosomes are apical to the nuclei. (C) Histochemistry section of Peyer's patch labelled with LAMP1 reveals the different morphology of the regular shaped apical FAE endosomes (red filled arrows) and the more pleiomorphic endosomes of SED macrophages, two of which seem to intrude into the FAE (black arrows). (D) EM micrograph of the large apical endosomes (arrows) of the FAE enterocytes located between the nuclei and the brush border. (E) The diameters of 100 LAMP1-positive endosomes in FAE enterocytes, villus enterocytes and M cells of 2 wild-type animals were measured. The average diameter of the LAMP1-positive endosomes of FAE enterocytes is significantly larger (p<0.05). Abbreviations: bb, brush border; G, goblet cells; ent, enterocyte; N, nucleus. Scale bars (A–C) 25 µm; (D) 750 nm.
(PDF)

Figure S2 **Distinguishing M cell and goblet cells by morphology and cellular markers.** (A) Typical M cells at FAE labelled with UEA-1 lectin. (B) goblet cells at the villi labelled with UEA-1. (C) Partial colocalization of M cell markers UEA-1 (red) and GP-2 (green) on FAE brush borders. (D) GP-2 (green) labels the brush borders of cells double labelled with a cytoplasmic M cell marker annexin V (AnxV, red) at FAE. Transmission EM micrographs of FAE reveal the clear morphological differences between M cells with typical short microvilli at their brush border (E), and the occasionally present goblet cells with their large apical mucus-containing secretory granules (F). Scale bars (A–D) 25 µm; (E–F) 500 nm.
(PDF)

Figure S3 **A high resolution version of** **Figure 1F** **of the original manuscript.** As with many cells active in water transport, FAE enterocytes and M cells have dilated intercellular spaces filled by interdigitating membrane leaflets. The microvilli of the M cells are heavily labelled with UEA-1 lectin (15 nm gold depicted with white arrows at the apical plasma membrane facing the intestinal lumen). In this micrograph some of the UEA-1 labelled leaflets (thick white arrows at the basolateral plasma membrane) of a M cell come close to the PrP positive endosomal vacuole labelled with (black arrow) in the cytoplasm of an FAE enterocyte and can be seen as a cross section. The dilated intercellular spaces and interdigitating membrane leaflets can be seen more clearly in Figure S5. PrP was detected with PrP-specific 6H4 monoclonal antibody directly conjugated to UltraSmall gold (PrP-usg) and visualized by silver enhancement.
(PDF)

Figure S4 **A high resolution version of** **Figure 2F** **of the original manuscript.** Boxed areas (a) and (b) in the left image are shown, right, at higher magnification. Black arrows indicate PrP-specific label between the apical microvilli facing the intestinal lumen (a) and within a multivesicular body (b). White arrowheads indicate LAMP1 labelling (15 nm gold) on the limiting membrane of these structures. PrP was detected with PrP-specific 6H4 monoclonal antibody directly conjugated to UltraSmall gold (PrP-usg) and visualized by silver enhancement.
(PDF)

Figure S5 **ME7 infected wt mice have detectable amounts of PrP-labeled brain inoculum in the gut lumen at 1 dpf.** A high resolution EM micrograph of the apical surface of an FAE enterocyte facing the gut lumen shows PrP-specific label (black arrows) in the lumen of an apical multivesicular body in boxed area (a) and in the gut lumen and between the microvilli facing the gut lumen in boxed area (b) better seen in the enlarged images below. PrP was detected with PrP-specific 6H4 monoclonal antibody directly conjugated to UltraSmall gold (PrP-usg) and visualized by silver enhancement. The section was double labelled with late endosomal marker LAMP-1, which was detected with protein A conjugated to 15 nm gold (white triangles) present on the limiting membrane of the endosome. Note the dilated intercellular spaces filled by interdigitating membrane leaflets. Scale bar 300 nm.
(PDF)

Figure S6 **Oral administration of FVB (wt) and Prnp^-/-^ mice with PK-treated Sc237 SHa brain homogenate.** Briefly, for oral infection via gavage 6 FVB (wt) and 6 *Prnp^-/-^* mice were exposed to PK-treated Sc237 Syrian hamster (SHa) brain homogenate. Three animals of both groups were sacrificed by cervical dislocation after 6 hours and the rest at 24 hours post infection. Animals were dissected and intestine was cut into 2–3 cm pieces and immersion-fixed in 2% PFA + 0.2% GA in PHEM buffer and processed for IEM as described in the Materials and methods. For controls 2 FVB and 2 *Prnp^-/-^* mice were exposed to PK-treated normal SHa brain homogenate. These animals were sacrificed after 6 and 24 hours after exposure and processed identically to the others. Brain homogenates (20% w/v) were digested with proteinase K (PK, 50 µg/ml at 37°C for 1 hour) in the presence of 2% Sarkosyl. The digestion was stopped by addition of 1 mM PMSF. Afterwards the brain homogenates were diluted to 10%, effectively reducing the Sarkosyl concentration to 1% and used in the oral exposure experiments. Prior to their use the PK-treated SHa brain homogenates (Sc237-infected and uninfected) were tested by Western blotting (using mAb 3F4) to confirm the complete digestion of PrP^C^ and the truncation of PrP^Sc^ to the PK-resistant PrP 27–30 core, respectively. (A) Western blot analysis of the hamster brain homogenates used for the mouse oral exposure experiment. Lanes 1 and 2 were loaded with uninfected Syrian hamster brain homogenate with or without PK treatment, respectively. Lanes 3 and 4 were loaded with brain homogenate from Sc237-infected Syrian hamsters with and without PK treatment, respectively. Quantitation of 3F4 positive endosomes in wt- mice (B) and *Prnp* ^-/-^ mice (C) 6 and 24 hours after exposure to PK-treated Sc237 SHa brain homogenate. For quantitation approximately 300 endosomes of FAE enterocytes (FAE), M cells (M) and villus enterocytes (E) were counted for each animal. Each bar represents percentage of 3F4 positive endosomes at given time point in total and gives the ratio of 3F4-positive electron-lucent early endosomal structures (EE) compared to 3F4-positive multivesicular bodies (MVB). For IEM analysis the Peyer's patch most proximal to the stomach of each animal was examined. The FAE and SED regions were immunostained using biotinylated mAb 3F4 (Covance Research Products). mAb 3F4 recognizes only PK-treated hamster PrP^Sc^. To exclude interference from endogenous immunoglobulins mAb 3F4 was conjugated directly to Streptavidin Gold Nanoparticles with 10 nm gold (Nanocs).
(PDF)

Figure S7 **PK-treated hamster brain-derived prion inoculum is found in early endosomes and multivesicular bodies of FAE enterocytes.** Electron micrographs reveal hamster PrP-specific labelling in small electron-lucent early endosomes at 6 h (A and B) and in multivesicular bodies at 24 h (C and D) after exposure to PK-treated Sc237 SHa brain homogenate. Sections of the most proximal Peyer's patch were labelled with mAb 3F4 directly conjugated to 10 nm gold. A and D are sections of wt mice and B and C from *Prnp* ^-/-^ mice, respectively. Similar PrP-positive endosomes were observed in oral exposure experiments with PrP-infected mouse brain homogenate (see Figure 2 in the main article).
(PDF)

Figure S8 **Microarray analysis of expression of ferritin light chain, major histocompatibility complex class II invariant chain and CD11c in macrophages and dendritic cells of different tissue-specific origin.** Analysis of 304 individual microarray data sets representing 1: bone marrow; 2: bone marrow progenitors; 3: bone marrow-derived macrophages; 4: peritoneal macrophages; 5: osteoclasts, microglia, Langerhans cells; 6: bone marrow-derived dendritic cells; 7: splenic, Peyer's patch and lymph node dendritic cells; 8: plasmacytoid dendritic cells; 9: natural killer cells/ IFN-producing killer dendritic cells; 10: myeloblasts and neutrophils; 11: B cells; 12: T cells. Adapted from Mabbott *et al.* (2010) Immunobiology 215:724-736.
(PDF)

Figure S9 **SED macrophages uptake prion inoculum.** (A) and (B) The PrP-containing brain inoculum is found in late endosomes of SED macrophages beneath the FAE at 1 dpf. The PrP (small black arrows) can be seen more clearly in the enlarged images (a) and (b) It was detected with PrP-specific 6H4 monoclonal antibody directly conjugated to UltraSmall gold (PrP-usg) and visualized by silver enhancement. The section was double labelled with MHC class II antibody, which was detected with protein A conjugated to 15 nm gold (small white arrows in the blow ups). In accordance with immunofluorescence data in main manuscript Figure 4 the phagocytes that engulf PrP inoculum express low levels of MHC class II (marker of antigen presenting cells/ classical dendritic cells) on their surface. Note also the extracellular exosome–like vesicles that are being either exocytosed or phagocytosed by the macrophage (big black arrow). Scale bars in (A) and (B) 200 nm; in enlarged inserts (a) and (b) 100 nm.
(PDF)

Figure S10 **Peyer's patch of ME7 infected wt mice at 105 dpf.** (A) and (B) Immunofluorescence detection of PrP-specific label in the germinal centres. In (A) the cryo section was labeled with rabbit polyclonal 1B3 and detected with goat anti-rabbit conjugated to Texas Red. In (B) section was double labelled with 1B6 (red) and rat monoclonal LAMP-1 (green). PrP is found on the plasma membrane of FDC and LAMP-1 positive late endosomes/lysosomes of tingible body macrophages (TBMs, white arrow in (B). Cryo immunogold EM reveals large accumulations of PrP upon FDC plasma membranes (C) and TBM late endosomes/lysosomes (D). The EM sections were labelled with R2-antibody directly conjugated to UltraSmall gold (PrP-usg) and visualized by silver enhancement. (E) After trypsin treatment an average of 18% of the PrP-specific label (white bar; scrapie +T) remains on FDC plasma membranes of PrP-infected mice when compared to untreated tissue sections from PrP-infected mice (black bar; scrapie –T; 100%). The remaining PrP after trypsin treatment is indicative for protease-resistant disease-related PrP^Sc^. Untreated (black bar, control -T) and trypsin-treated (grey bar, control +T) uninfected mice show little PrP-specific label. Samples were collected at 105 dpf and the FDC plasma membrane bound PrP-specific gold was counted in untreated and trypsin treated Peyer's patch cryosections of ME7 infected mice and noninfected controls. Ten mature FDC cells in the germinal centre were randomly selected and 50 µm of plasma membrane was analysed per cell. A total membrane length of 500 µm per treatment was analysed and the result is given as a relative labeling density per membrane length ± SD. (F) After trypsin treatment an average of 56% of the PrP-specific label (white bar; scrapie +T) remains in the lumen of late endosomes/lysosomes of PrP-infected mice when compared to untreated sections from PrP-infected mice (black bar; scrapie –T; 100%). The remaining PrP after trypsin treatment is indicative for protease-resistant forms of disease-related PrP^Sc^. Untreated (black bar,control -T) and trypsin-treated (grey bar, control +T) uninfected mice show little late endosome/lysosome associated PrP-specific label. Samples were collected at 105 dpf and the PrP-specific gold was counted in the lumen of late endosomes/lysosomes of tingible body macrophages at the germinal centre of untreated and trypsin treated Peyer's patch cryosections of ME7 infected mice and noninfected controls. A total of 100 late endosomes/lysosomes per treatment was analysed and the result is given as a relative labellng density per area ± SED. Scale bars (A and B) 25 µm and (C and D) 200 nm.
(PDF)

Figure S11 **The number of PrP-positive late endosomes in TBMs increased during the early course of prion infection.** Quantification was performed on Peyer's patches of 2 ME7-infected, wt mice at the time-points indicated (x axis). Percentage of TBMs with PrP- and LAMP1-positive endosomes in germinal centres are indicated relative to the total TBMs observed. A total area of 100 µm^2^/animal in the germinal centre was analysed.
(PDF)

Figure S12 **Expression of Lamp1, Gpa33 and Prnp mRNA in murine tissues and cells.** (A) Gene expression analysed using the Reference Database of Immune Cell types (RefDIC; Hijikata et al. (2007) Bioinformatics 23, 2934-2941). Detailed conditions of cell and tissue preparation and treatment are available on the RefDIC website (http://refdic.rcai.riken.jp/welcome.cgi). mRNA expression was analyzed in different cell types from different organs, as indicated by the top row and corresponding key. Normalised expression level data for each gene are shown (second to forth rows) as a gradation from green (low) to red (high). DC, dendritic cells; F, follicle-associated epithelium; FDC, follicular dendritic cell; V, villus epithelium; Mast, mast cells; NK, NK cells; NKT, Natural killer T cells; Neut, neutrophil; Lk, Peyer's patch leukocytes. (B) Expression profile of *Gpa33* in 61 murine tissues and cells analysed using the GNF1M Mouse tissue atlas (Su, et al. (2004) Proc. Natl. Acad. Sci. USA 101, 6062-6067). Detailed conditions of the cell and tissue preparation and treatment are available in the above study. Normalised gene expression level data for each tissue and cell type are shown.
(PDF)

Figure S13 **Labeling of A33 in Peyer's patches of wild-type and A33^-/-^ mice.** Immunofluorescence analysis of Peyer's patches shows A33 antigen (red) at the FAE and villus epithelium of the wild-type (A) mouse, but not in the A33^-/-^ mouse (B). Two separate Peyer's patches of wt and A33-/- mice were analyzed. The LAMP1 label (green) shows the location of FAE and SED. (C) Quantification of the A33 immunogold labeling density in electron micrographs of Peyer's patches of wild-type and A33^-/-^ mice. The total number of protein A–gold (PAG) particles were counted on 100 µm^2^ of germinal centres from 2 mice and then averaged. Equal areas were counted in control sections that were exposed to PAG only and the A33 antibody was omitted. Scale bar in (A) and (B): 25 µm.
(PDF)
