# Supplementary figures and images for: Prion Uptake in the Gut: Identification of the First Uptake and Replication Sites
Source: PLoS Pathog. 2011 Dec 22;7(12):e1002449. doi: 10.1371/journal.ppat.1002449 (PMC3245311; doi:10.1371/journal.ppat.1002449)

# Supplementary data

Fig. S1

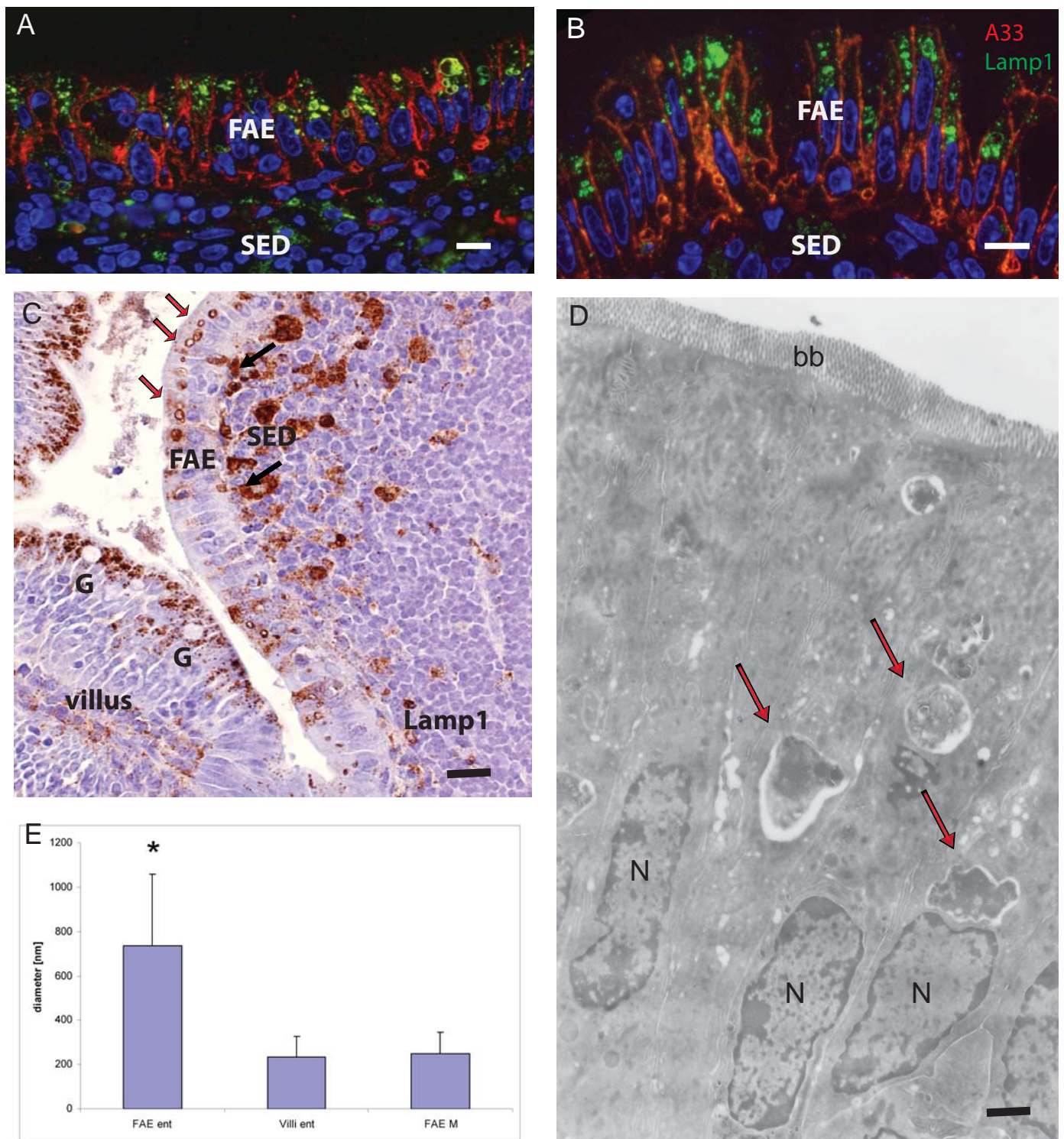

Supplement: Figure S1 — FAE enterocytes have larger late endosomes than enterocytes in the neigbouring villi or M cells in FAE. (A) and (B) show IF images of FAE double labelled with epithelium-specific A33 (red) and late endosomal marker LAMP1 (green). Nuclei are labelled with Dapi (blue). The large FAE endosomes are apical to the nuclei. (C) Histochemistry section of Peyer's patch labelled with LAMP1 reveals the different morphology of the regular shaped apical FAE endosomes (red filled arrows) and the more pleiomorphic endosomes of SED macrophages, two of which seem to intrude into the FAE (black arrows). (D) EM micrograph of the large apical endosomes (arrows) of the FAE enterocytes located between the nuclei and the brush border. (E) The diameters of 100 LAMP1-positive endosomes in FAE enterocytes, villus enterocytes and M cells of 2 wild-type animals were measured. The average diameter of the LAMP1-positive endosomes of FAE enterocytes is significantly larger (p<0.05). Abbreviations: bb, brush border; G, goblet cells; ent, enterocyte; N, nucleus. Scale bars (A–C) 25 µm; (D) 750 nm. (PDF) [file ppat.1002449.s001.pdf]

FIG. S2

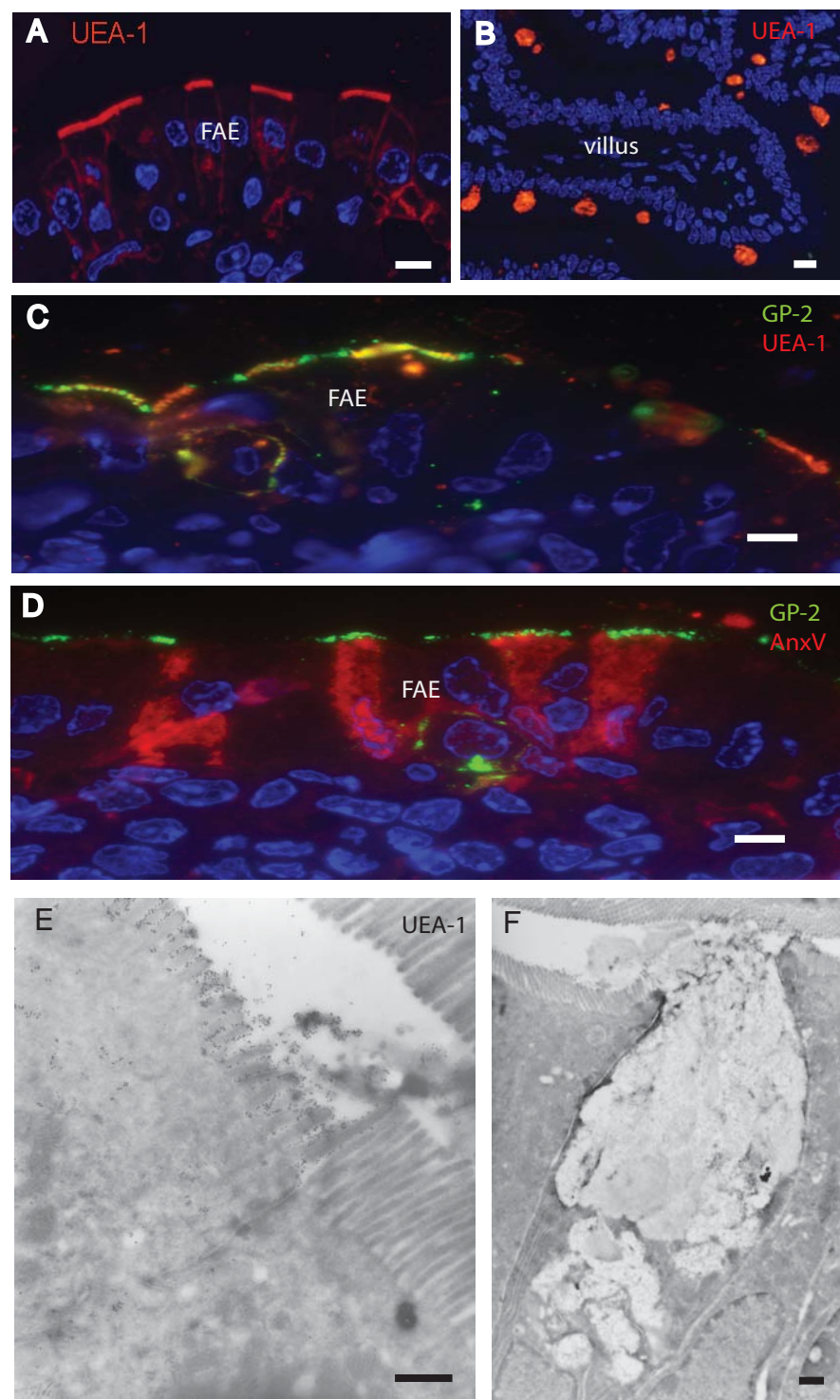

Supplement: Figure S2 — Distinguishing M cell and goblet cells by morphology and cellular markers. (A) Typical M cells at FAE labelled with UEA-1 lectin. (B) goblet cells at the villi labelled with UEA-1. (C) Partial colocalization of M cell markers UEA-1 (red) and GP-2 (green) on FAE brush borders. (D) GP-2 (green) labels the brush borders of cells double labelled with a cytoplasmic M cell marker annexin V (AnxV, red) at FAE. Transmission EM micrographs of FAE reveal the clear morphological differences between M cells with typical short microvilli at their brush border (E), and the occasionally present goblet cells with their large apical mucus-containing secretory granules (F). Scale bars (A–D) 25 µm; (E–F) 500 nm. (PDF) [file ppat.1002449.s002.pdf]

Fig. S3

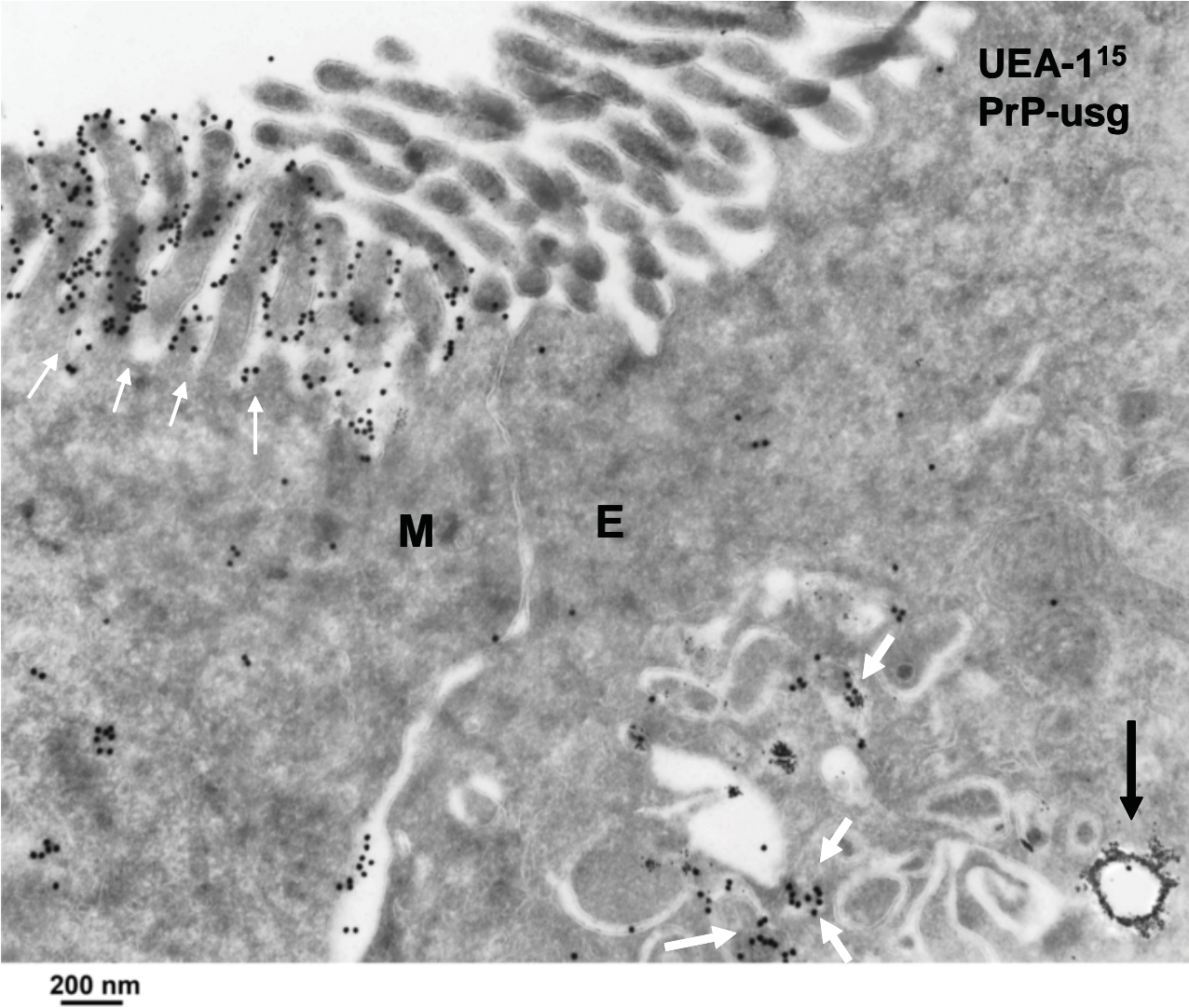

Supplement: Figure S3 — A high resolution version of Figure 1F of the original manuscript. As with many cells active in water transport, FAE enterocytes and M cells have dilated intercellular spaces filled by interdigitating membrane leaflets. The microvilli of the M cells are heavily labelled with UEA-1 lectin (15 nm gold depicted with white arrows at the apical plasma membrane facing the intestinal lumen). In this micrograph some of the UEA-1 labelled leaflets (thick white arrows at the basolateral plasma membrane) of a M cell come close to the PrP positive endosomal vacuole labelled with (black arrow) in the cytoplasm of an FAE enterocyte and can be seen as a cross section. The dilated intercellular spaces and interdigitating membrane leaflets can be seen more clearly in Figure S5. PrP was detected with PrP-specific 6H4 monoclonal antibody directly conjugated to UltraSmall gold (PrP-usg) and visualized by silver enhancement. (PDF) [file ppat.1002449.s003.pdf]

Fig. S4

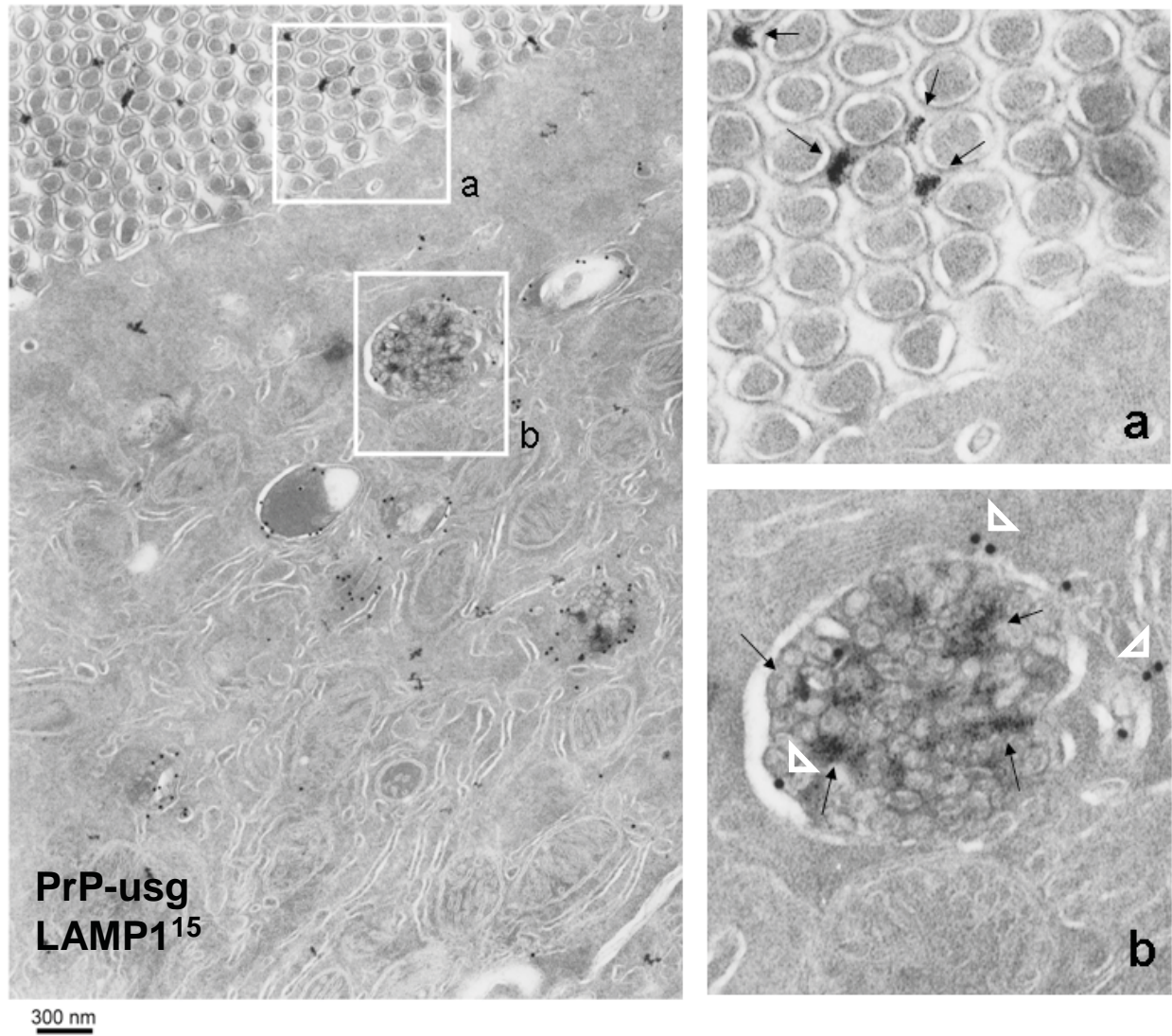

Supplement: Figure S4 — A high resolution version of Figure 2F of the original manuscript. Boxed areas (a) and (b) in the left image are shown, right, at higher magnification. Black arrows indicate PrP-specific label between the apical microvilli facing the intestinal lumen (a) and within a multivesicular body (b). White arrowheads indicate LAMP1 labelling (15 nm gold) on the limiting membrane of these structures. PrP was detected with PrP-specific 6H4 monoclonal antibody directly conjugated to UltraSmall gold (PrP-usg) and visualized by silver enhancement. (PDF) [file ppat.1002449.s004.pdf]

Fig. S5

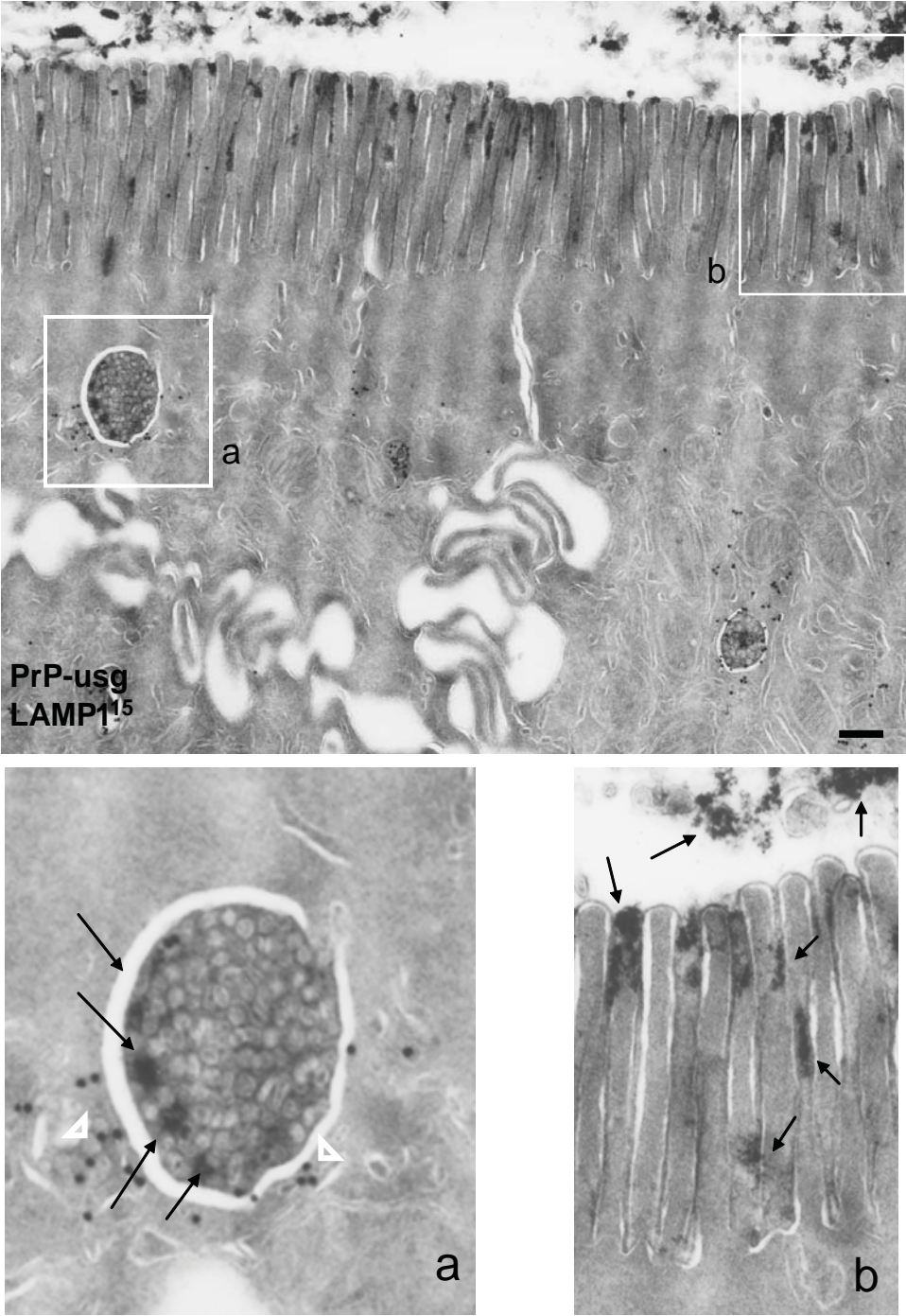

Supplement: Figure S5 — ME7 infected wt mice have detectable amounts of PrP-labeled brain inoculum in the gut lumen at 1 dpf. A high resolution EM micrograph of the apical surface of an FAE enterocyte facing the gut lumen shows PrP-specific label (black arrows) in the lumen of an apical multivesicular body in boxed area (a) and in the gut lumen and between the microvilli facing the gut lumen in boxed area (b) better seen in the enlarged images below. PrP was detected with PrP-specific 6H4 monoclonal antibody directly conjugated to UltraSmall gold (PrP-usg) and visualized by silver enhancement. The section was double labelled with late endosomal marker LAMP-1, which was detected with protein A conjugated to 15 nm gold (white triangles) present on the limiting membrane of the endosome. Note the dilated intercellular spaces filled by interdigitating membrane leaflets. Scale bar 300 nm. (PDF) [file ppat.1002449.s005.pdf]

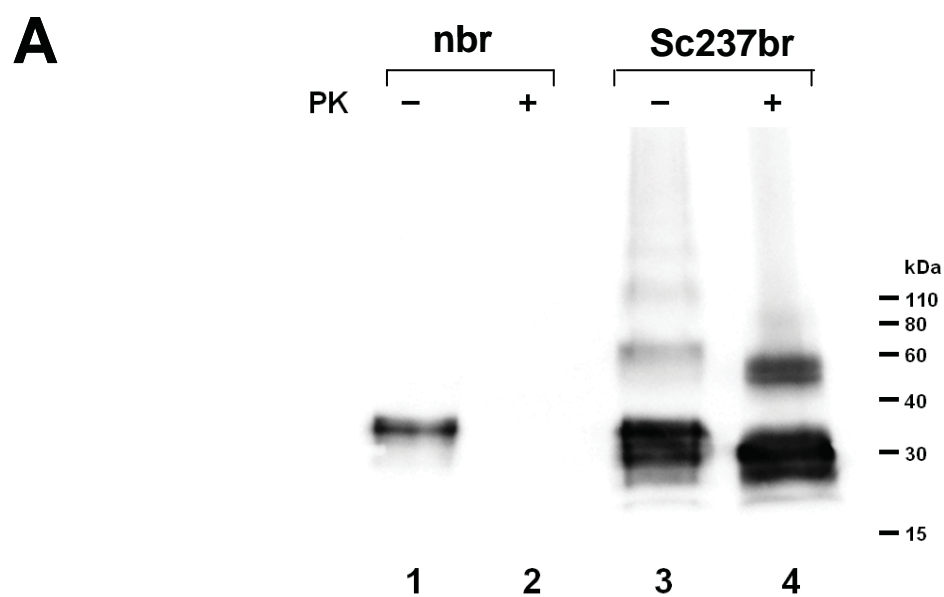

**B**

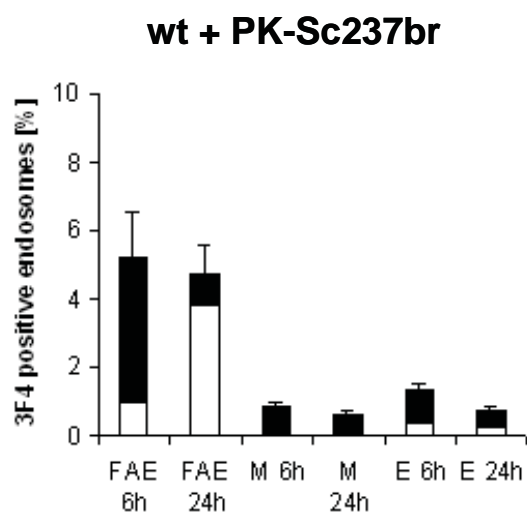

**C**

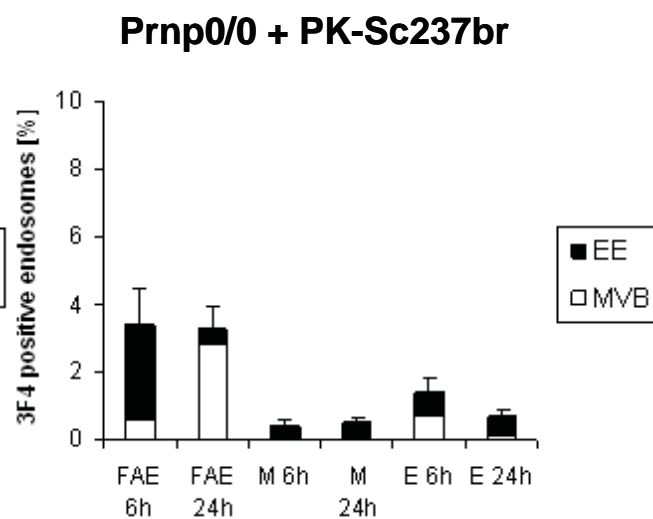

Fig. S6

Supplement: Figure S6 — Oral administration of FVB (wt) and Prnp-/- mice with PK-treated Sc237 SHa brain homogenate. Briefly, for oral infection via gavage 6 FVB (wt) and 6 Prnp-/- mice were exposed to PK-treated Sc237 Syrian hamster (SHa) brain homogenate. Three animals of both groups were sacrificed by cervical dislocation after 6 hours and the rest at 24 hours post infection. Animals were dissected and intestine was cut into 2–3 cm pieces and immersion-fixed in 2% PFA + 0.2% GA in PHEM buffer and processed for IEM as described in the Materials and methods. For controls 2 FVB and 2 Prnp-/- mice were exposed to PK-treated normal SHa brain homogenate. These animals were sacrificed after 6 and 24 hours after exposure and processed identically to the others. Brain homogenates (20% w/v) were digested with proteinase K (PK, 50 µg/ml at 37°C for 1 hour) in the presence of 2% Sarkosyl. The digestion was stopped by addition of 1 mM PMSF. Afterwards the brain homogenates were diluted to 10%, effectively reducing the Sarkosyl concentration to 1% and used in the oral exposure experiments. Prior to their use the PK-treated SHa brain homogenates (Sc237-infected and uninfected) were tested by Western blotting (using mAb 3F4) to confirm the complete digestion of PrPC and the truncation of PrPSc to the PK-resistant PrP 27–30 core, respectively. (A) Western blot analysis of the hamster brain homogenates used for the mouse oral exposure experiment. Lanes 1 and 2 were loaded with uninfected Syrian hamster brain homogenate with or without PK treatment, respectively. Lanes 3 and 4 were loaded with brain homogenate from Sc237-infected Syrian hamsters with and without PK treatment, respectively. Quantitation of 3F4 positive endosomes in wt- mice (B) and Prnp -/- mice (C) 6 and 24 hours after exposure to PK-treated Sc237 SHa brain homogenate. For quantitation approximately 300 endosomes of FAE enterocytes (FAE), M cells (M) and villus enterocytes (E) were counted for each animal. Each bar represents percentage o [file ppat.1002449.s006.pdf]

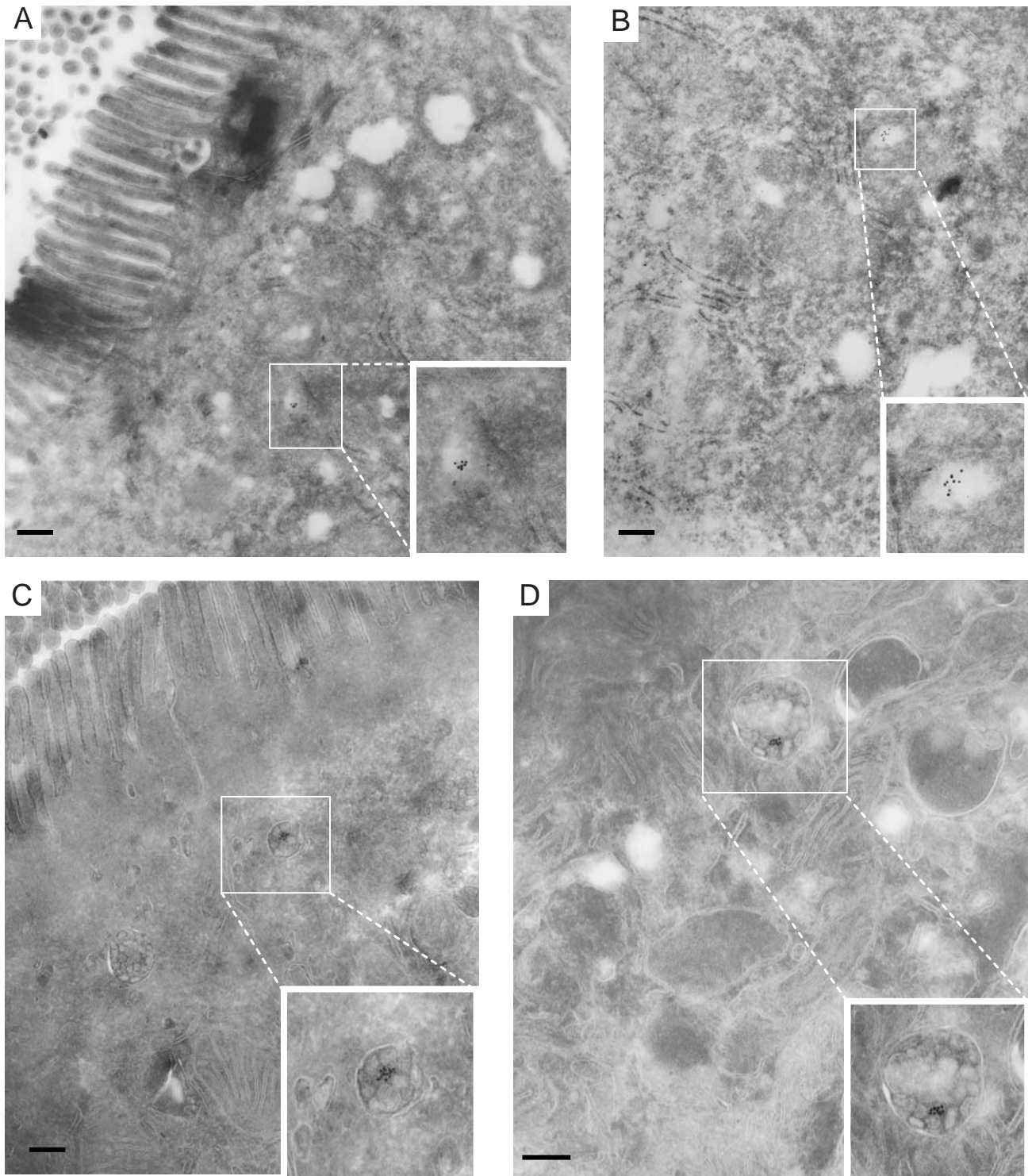

Fig. S7

Supplement: Figure S7 — PK-treated hamster brain-derived prion inoculum is found in early endosomes and multivesicular bodies of FAE enterocytes. Electron micrographs reveal hamster PrP-specific labelling in small electron-lucent early endosomes at 6 h (A and B) and in multivesicular bodies at 24 h (C and D) after exposure to PK-treated Sc237 SHa brain homogenate. Sections of the most proximal Peyer's patch were labelled with mAb 3F4 directly conjugated to 10 nm gold. A and D are sections of wt mice and B and C from Prnp -/- mice, respectively. Similar PrP-positive endosomes were observed in oral exposure experiments with PrP-infected mouse brain homogenate (see Figure 2 in the main article). (PDF) [file ppat.1002449.s007.pdf]

Fig. S9

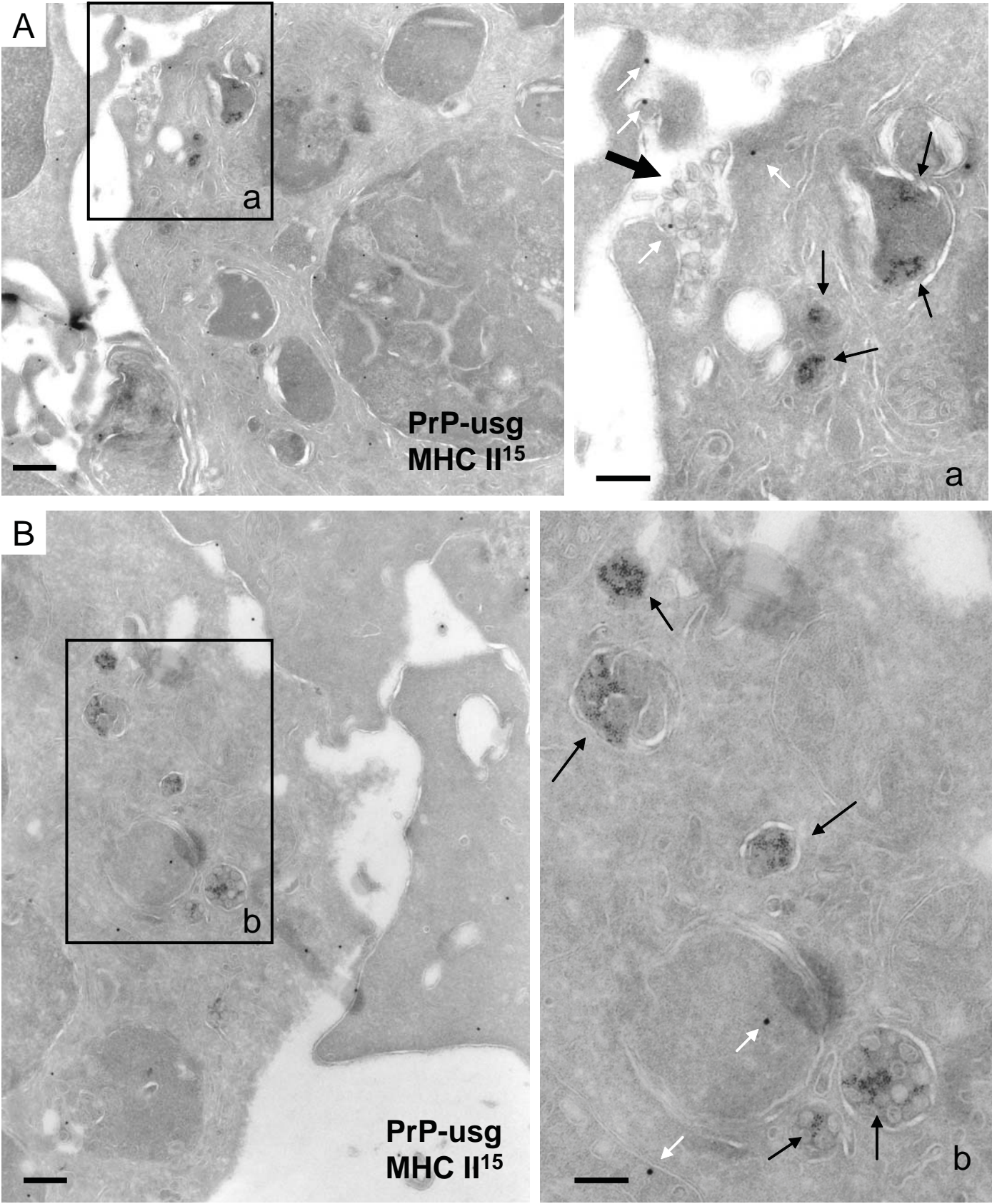

Supplement: Figure S9 — SED macrophages uptake prion inoculum. (A) and (B) The PrP-containing brain inoculum is found in late endosomes of SED macrophages beneath the FAE at 1 dpf. The PrP (small black arrows) can be seen more clearly in the enlarged images (a) and (b) It was detected with PrP-specific 6H4 monoclonal antibody directly conjugated to UltraSmall gold (PrP-usg) and visualized by silver enhancement. The section was double labelled with MHC class II antibody, which was detected with protein A conjugated to 15 nm gold (small white arrows in the blow ups). In accordance with immunofluorescence data in main manuscript Figure 4 the phagocytes that engulf PrP inoculum express low levels of MHC class II (marker of antigen presenting cells/ classical dendritic cells) on their surface. Note also the extracellular exosome–like vesicles that are being either exocytosed or phagocytosed by the macrophage (big black arrow). Scale bars in (A) and (B) 200 nm; in enlarged inserts (a) and (b) 100 nm. (PDF) [file ppat.1002449.s009.pdf]

Fig. S10

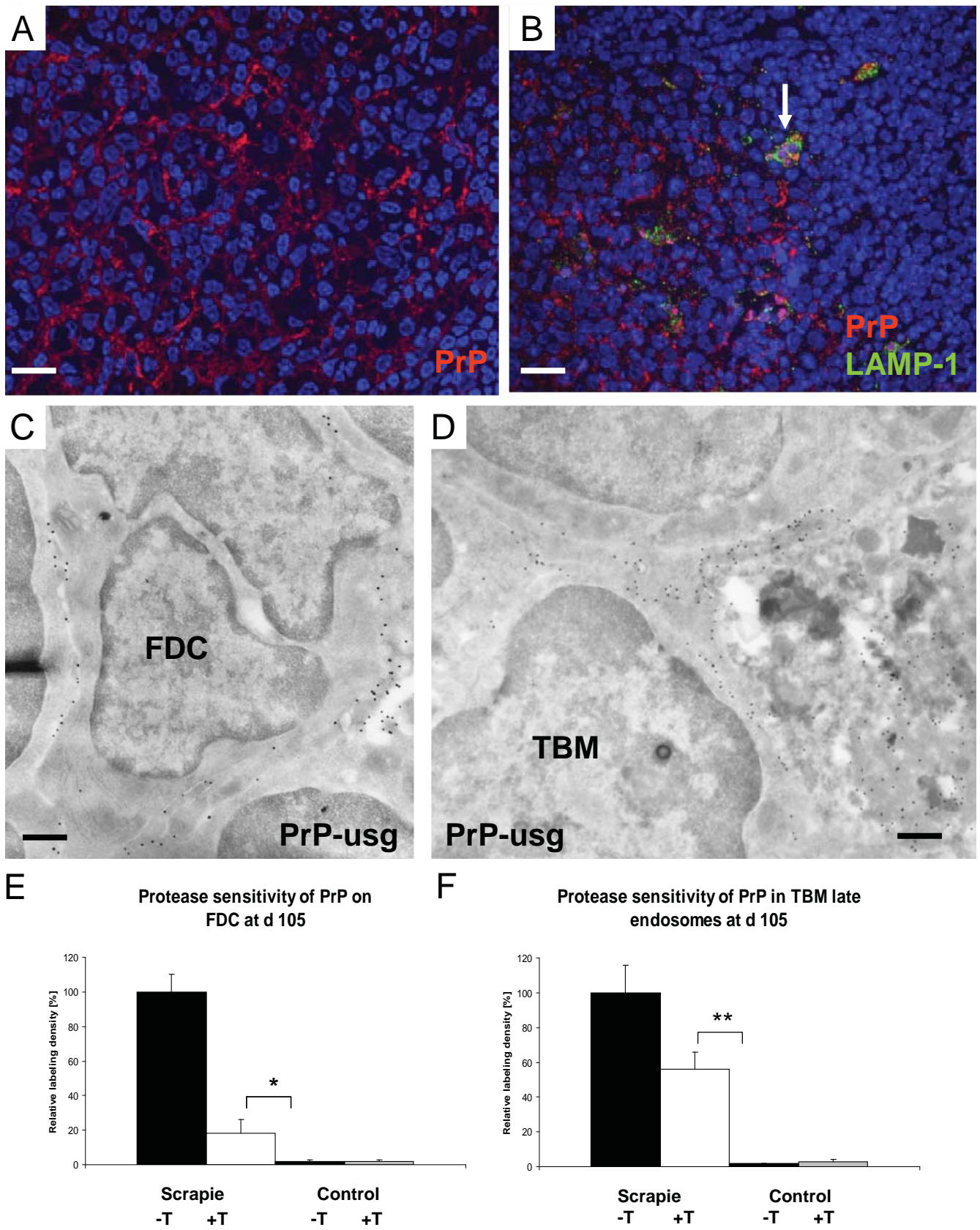

Supplement: Figure S10 — Peyer's patch of ME7 infected wt mice at 105 dpf. (A) and (B) Immunofluorescence detection of PrP-specific label in the germinal centres. In (A) the cryo section was labeled with rabbit polyclonal 1B3 and detected with goat anti-rabbit conjugated to Texas Red. In (B) section was double labelled with 1B6 (red) and rat monoclonal LAMP-1 (green). PrP is found on the plasma membrane of FDC and LAMP-1 positive late endosomes/lysosomes of tingible body macrophages (TBMs, white arrow in (B). Cryo immunogold EM reveals large accumulations of PrP upon FDC plasma membranes (C) and TBM late endosomes/lysosomes (D). The EM sections were labelled with R2-antibody directly conjugated to UltraSmall gold (PrP-usg) and visualized by silver enhancement. (E) After trypsin treatment an average of 18% of the PrP-specific label (white bar; scrapie +T) remains on FDC plasma membranes of PrP-infected mice when compared to untreated tissue sections from PrP-infected mice (black bar; scrapie –T; 100%). The remaining PrP after trypsin treatment is indicative for protease-resistant disease-related PrPSc. Untreated (black bar, control -T) and trypsin-treated (grey bar, control +T) uninfected mice show little PrP-specific label. Samples were collected at 105 dpf and the FDC plasma membrane bound PrP-specific gold was counted in untreated and trypsin treated Peyer's patch cryosections of ME7 infected mice and noninfected controls. Ten mature FDC cells in the germinal centre were randomly selected and 50 µm of plasma membrane was analysed per cell. A total membrane length of 500 µm per treatment was analysed and the result is given as a relative labeling density per membrane length ± SD. (F) After trypsin treatment an average of 56% of the PrP-specific label (white bar; scrapie +T) remains in the lumen of late endosomes/lysosomes of PrP-infected mice when compared to untreated sections from PrP-infected mice (black bar; scrapie –T; 100%). The remaining PrP after trypsin treatment is indicative for [file ppat.1002449.s010.pdf]

Fig. S11

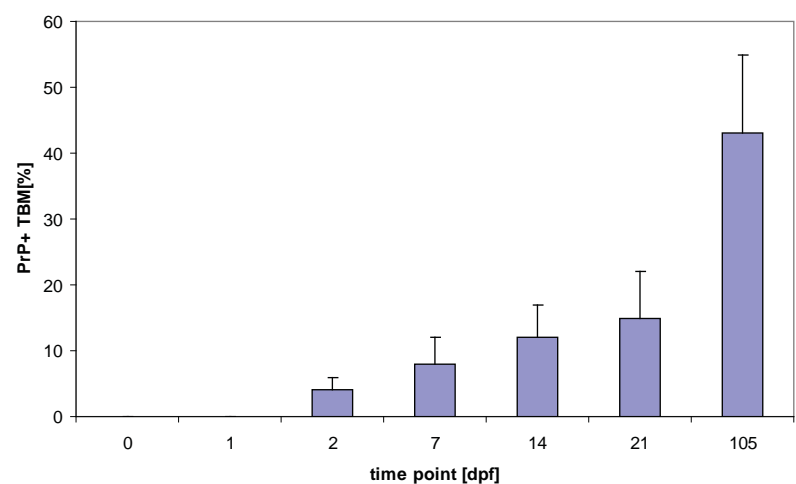

Supplement: Figure S11 — The number of PrP-positive late endosomes in TBMs increased during the early course of prion infection. Quantification was performed on Peyer's patches of 2 ME7-infected, wt mice at the time-points indicated (x axis). Percentage of TBMs with PrP- and LAMP1-positive endosomes in germinal centres are indicated relative to the total TBMs observed. A total area of 100 µm2/animal in the germinal centre was analysed. (PDF) [file ppat.1002449.s011.pdf]
