# Supplementary material for: Prion Uptake in the Gut: Identification of the First Uptake and Replication Sites
Source: PLoS Pathog. 2011 Dec 22;7(12):e1002449. doi: 10.1371/journal.ppat.1002449 (PMC3245311; doi:10.1371/journal.ppat.1002449)
Supplement: Figure S8 — Microarray analysis of expression of ferritin light chain, major histocompatibility complex class II invariant chain and CD11c in macrophages and dendritic cells of different tissue-specific origin. Analysis of 304 individual microarray data sets representing 1: bone marrow; 2: bone marrow progenitors; 3: bone marrow-derived macrophages; 4: peritoneal macrophages; 5: osteoclasts, microglia, Langerhans cells; 6: bone marrow-derived dendritic cells; 7: splenic, Peyer's patch and lymph node dendritic cells; 8: plasmacytoid dendritic cells; 9: natural killer cells/ IFN-producing killer dendritic cells; 10: myeloblasts and neutrophils; 11: B cells; 12: T cells. Adapted from Mabbott et al. (2010) Immunobiology 215:724-736. (PDF) [file ppat.1002449.s008.pdf]

Ferritin light chain (*Ftl1*) is expressed at high levels by macrophages

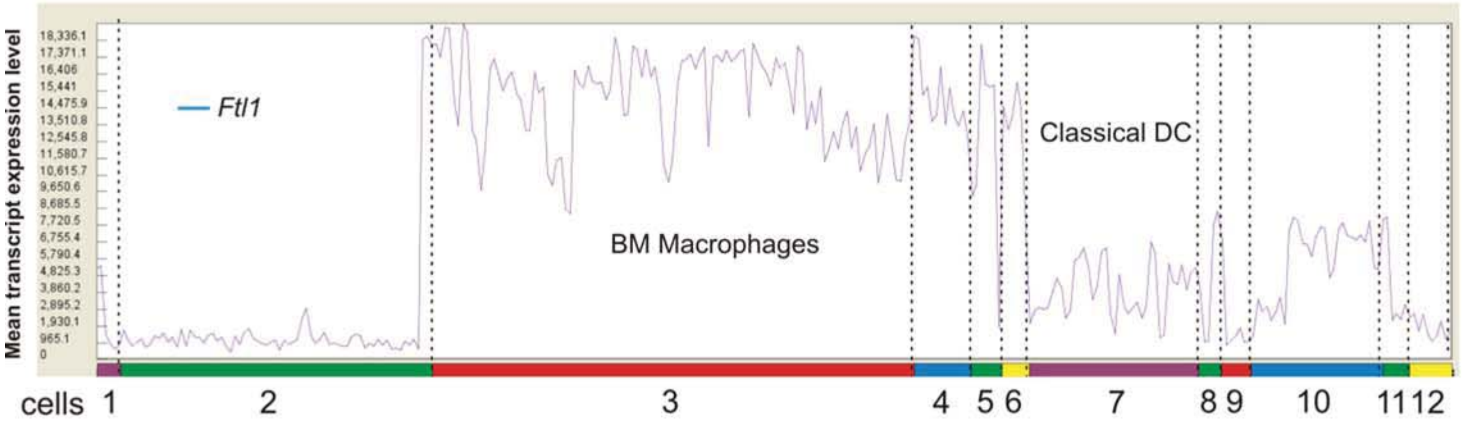

Major histocompatibility complex class II invariant chain (*Cd74*) is expressed at high levels by classical DC

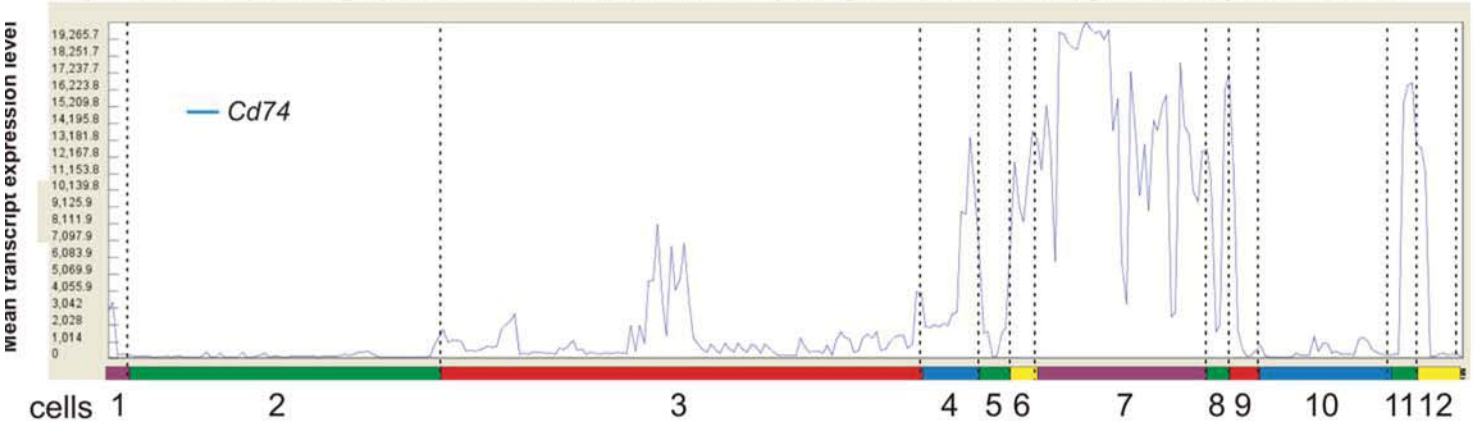

CD11c (*Itgax*) is expressed at high levels by classical DC

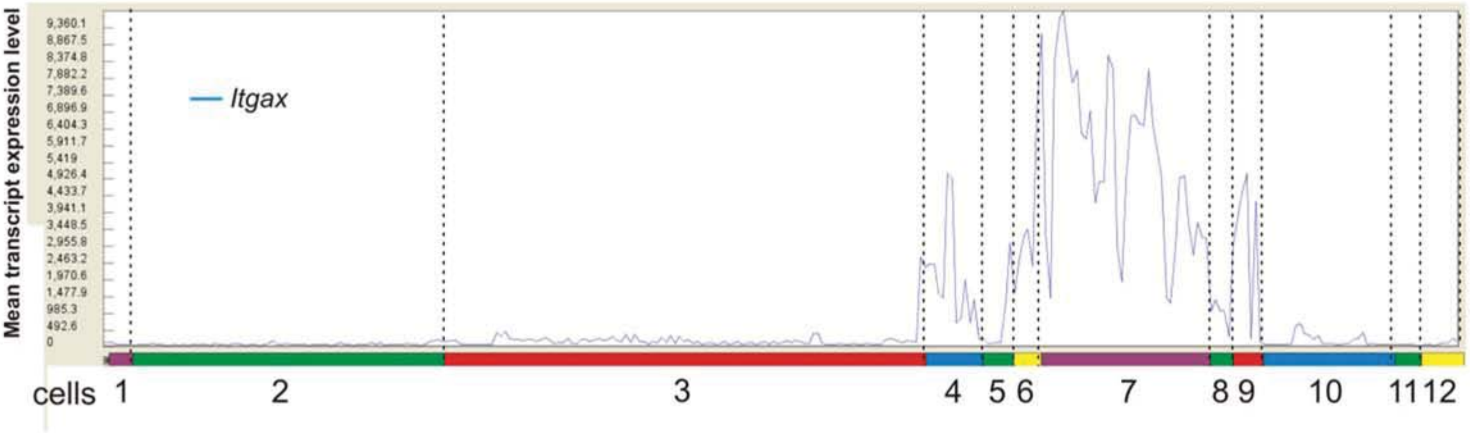

Fig. S8
