# Supplementary material for: Prion Uptake in the Gut: Identification of the First Uptake and Replication Sites
Source: PLoS Pathog. 2011 Dec 22;7(12):e1002449. doi: 10.1371/journal.ppat.1002449 (PMC3245311; doi:10.1371/journal.ppat.1002449)
Supplement: Figure S13 — Labeling of A33 in Peyer's patches of wild-type and A33-/- mice. Immunofluorescence analysis of Peyer's patches shows A33 antigen (red) at the FAE and villus epithelium of the wild-type (A) mouse, but not in the A33-/- mouse (B). Two separate Peyer's patches of wt and A33-/- mice were analyzed. The LAMP1 label (green) shows the location of FAE and SED. (C) Quantification of the A33 immunogold labeling density in electron micrographs of Peyer's patches of wild-type and A33-/- mice. The total number of protein A–gold (PAG) particles were counted on 100 µm2 of germinal centres from 2 mice and then averaged. Equal areas were counted in control sections that were exposed to PAG only and the A33 antibody was omitted. Scale bar in (A) and (B): 25 µm. (PDF) [file ppat.1002449.s013.pdf]

Fig. S13

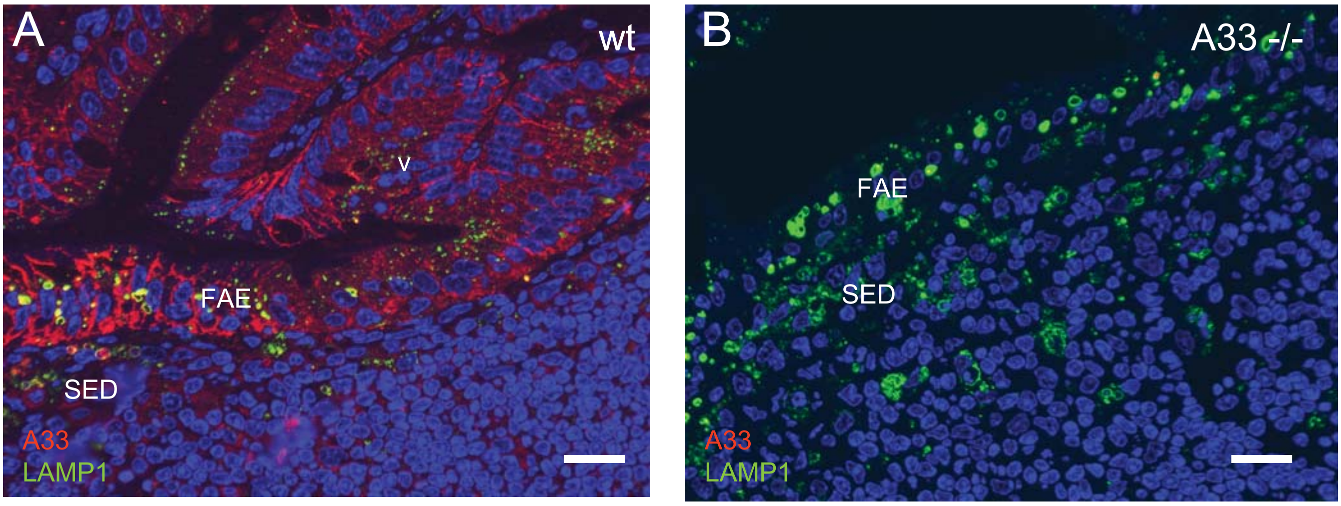

C Label (gold average per 100 µm2 ± SD) wild type and A33 -/- mice in germinal centres.

	anti-A33+ PAG	PAG only
wt	112 ± 11	8 ± 4
A33 -/-	11 ± 5	12 ± 6
