# Supplementary material for: Prion Uptake in the Gut: Identification of the First Uptake and Replication Sites
Source: PLoS Pathog. 2011 Dec 22;7(12):e1002449. doi: 10.1371/journal.ppat.1002449 (PMC3245311; doi:10.1371/journal.ppat.1002449)
Supplement: Figure S12 — Expression of Lamp1, Gpa33 and Prnp mRNA in murine tissues and cells. (A) Gene expression analysed using the Reference Database of Immune Cell types (RefDIC; Hijikata et al. (2007) Bioinformatics 23, 2934-2941). Detailed conditions of cell and tissue preparation and treatment are available on the RefDIC website (http://refdic.rcai.riken.jp/welcome.cgi). mRNA expression was analyzed in different cell types from different organs, as indicated by the top row and corresponding key. Normalised expression level data for each gene are shown (second to forth rows) as a gradation from green (low) to red (high). DC, dendritic cells; F, follicle-associated epithelium; FDC, follicular dendritic cell; V, villus epithelium; Mast, mast cells; NK, NK cells; NKT, Natural killer T cells; Neut, neutrophil; Lk, Peyer's patch leukocytes. (B) Expression profile of Gpa33 in 61 murine tissues and cells analysed using the GNF1M Mouse tissue atlas (Su, et al. (2004) Proc. Natl. Acad. Sci. USA 101, 6062-6067). Detailed conditions of the cell and tissue preparation and treatment are available in the above study. Normalised gene expression level data for each tissue and cell type are shown. (PDF) [file ppat.1002449.s012.pdf]

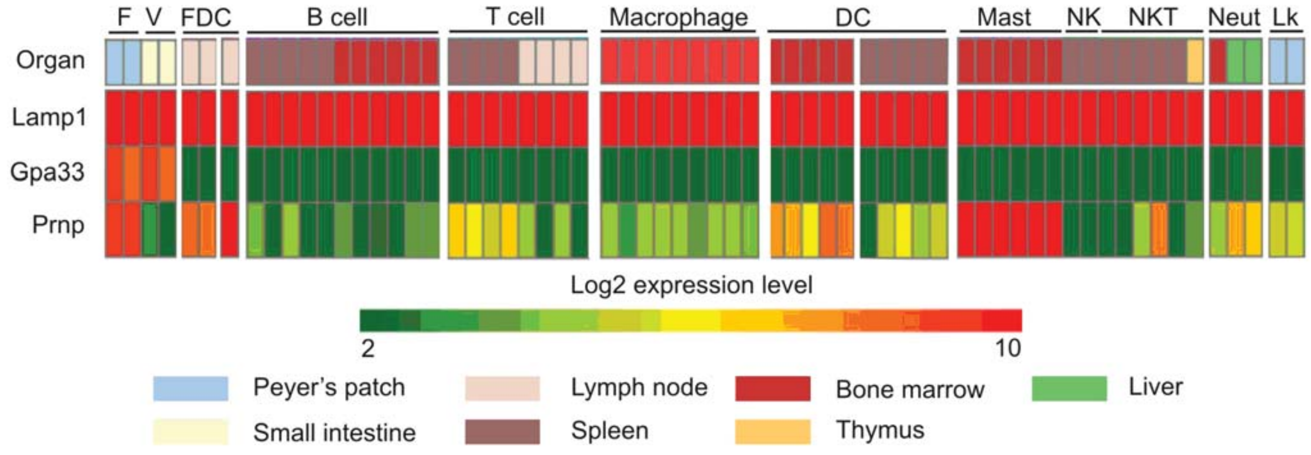

Fig. S12A

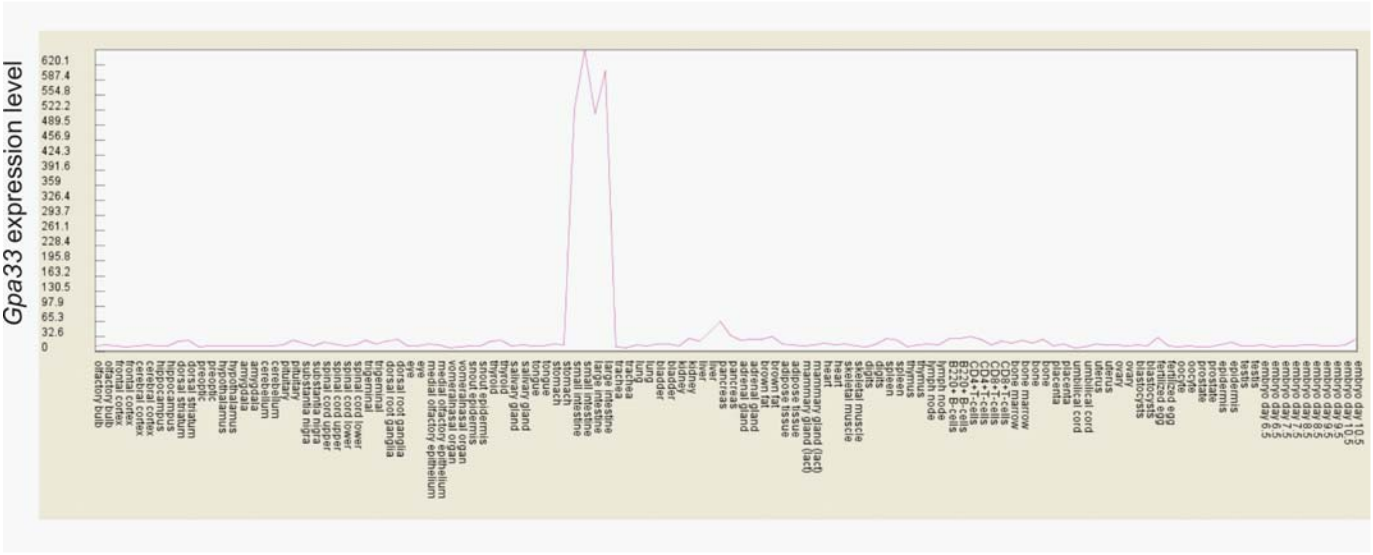

Fig. S12B
